# Expression Profile of Sonic Hedgehog Pathway Members in the Developing Human Fetal Brain

**DOI:** 10.1155/2015/494269

**Published:** 2015-07-21

**Authors:** Julia Tichy, Jenny Zinke, Benedikt Bunz, Richard Meyermann, Patrick N. Harter, Michel Mittelbronn

**Affiliations:** ^1^Institute of Pathology and Neuropathology, University of Tuebingen, 72076 Tuebingen, Germany; ^2^Senckenberg Institute for Neurooncology, Goethe University Frankfurt, 60528 Frankfurt am Main, Germany; ^3^Edinger Institute, Institute of Neurology, Goethe University Frankfurt, 60528 Frankfurt am Main, Germany

## Abstract

The Sonic Hedgehog (*SHH*) pathway plays a central role in the developing mammalian CNS. In our study, we aimed to investigate the spatiotemporal *SHH* pathway expression pattern in human fetal brains. We analyzed 22 normal fetal brains for Shh, Patched, Smoothened, and Gli1-3 expression by immunohistochemistry. In the telencephalon, strongest expression of Shh, Smoothened, and Gli2 was found in the cortical plate (CP) and ventricular zone. Patched was strongly upregulated in the ventricular zone and Gli1 in the CP. In the cerebellum, *SHH* pathway members were strongly expressed in the external granular layer (EGL). *SHH* pathway members significantly decreased over time in the ventricular and subventricular zone and in the cerebellar EGL, while increasing levels were found in more superficial telencephalic layers. Our findings show that *SHH* pathway members are strongly expressed in areas important for proliferation and differentiation and indicate a temporal expression gradient in telencephalic and cerebellar layers probably due to decreased proliferation of progenitor cells and increased differentiation. Our data about the spatiotemporal expression of *SHH* pathway members in the developing human brain serves as a base for the understanding of both normal and pathological CNS development.

## 1. Introduction

The Hedgehog (HH) pathway plays a central role in the embryonic and fetal development of both invertebrates and vertebrates [1]. This pathway essentially regulates cellular stemness, proliferation, apoptosis, migration, and differentiation [2–6]. While the hedgehog gene was first described in Drosophila melanogaster, its homologous genes in mammals meanwhile consist of the hedgehog family members Indian (*IHH*), Desert (*DHH*), and Sonic hedgehog (*SHH*) [7–9]. In general, the hedgehog proteins interact with the transmembrane receptor Patched (*PTCH*), the latter inhibiting the cascade-initiating receptor Smoothened (*SMO*) in the absence of hedgehog ligands [10]. The Patched-mediated inhibition of Smoothened is abolished in case of HH ligands binding to the Patched receptor [11]. The* SMO* activation elicits a nuclear translocation of the transcription factors Gli1, Gli2, and Gli3 [12]. Although the definite functions of the Gli family members are unclear, there is rising evidence for a predominantly transcription-activating function of Gli1 and Gli2 [13, 14]. In contrast, Gli3 is supposed to act more likely as an inhibitor of transcription [15]. While the hedgehog protein in Drosophila is a key player in the formation of segment polarity, its best characterized mammalian homologue Sonic hedgehog is involved in the development of the CNS, limbs, skeleton, and internal organs [16–20]. In the mammalian central nervous system (CNS)* SHH* is responsible for the patterning of the spinal cord and brain as well as for the proliferation of the cerebellar granule precursor cells [21]. Furthermore, it induces the differentiation of Bergmann glia in the developing cerebellum [21]. The importance of the* SHH* pathway for normal development and organ homeostasis is reflected by the fact that mutations in the* SHH* pathway lead to severe malformations and the development of malignant neoplasms [22, 23]. While pathogenic mutations of the* SHH* pathway among other diseases cause holoprosencephaly [20, 22], a deletion of the* Gli3* gene may result in the Greig cephalopolysyndactyly syndrome [24, 25]. On the other hand an increased activation of the* SHH* pathway may lead to neoplastic transformation of various tissues including breast [26], skin [27], and brain [21]. Most interestingly, neoplasms associated with mutations of the* SHH* pathway occur in areas where* SHH* is essential for the regulation of cellular stemness and differentiation such as in the cerebellum, the brain area where medulloblastomas emerge [28, 29]. Although the expression of the molecules of the* SHH* pathway in humans is very well studied under pathological conditions, there is a lack of knowledge concerning the spatiotemporal expression pattern in the normal developing human CNS. The aim of our study was to determine the nonpathological expression pattern of the* SHH* pathway members in a cohort of normal human fetal brains at different stages of gestation to gain insights into the role of the* SHH* pathway in normal CNS development.

## 2. Materials and Methods

### 2.1. Clinical Data and Tissue Specimens

We investigated 22 human fetal brains from the 12th to the 28th week of gestation (Table 1). For the present study a cohort of fetuses without pathological CNS alterations was selected according to clinical data, family history, and neuropathological analyses performed by two experienced neuropathologists (MM and RM). The samples belong to the human tissue brain bank of the Institute of Pathology and Neuropathology, University of Tuebingen, Germany [30]. All tissue specimens were paraffin-embedded. The study protocol was endorsed by the ethical committee of the University of Tuebingen, Germany.

### 2.2. Immunohistochemistry

All samples were fixed in 4% formalin (pH 7.4) and embedded in paraffin. Paraffin blocks were cut and sections of 4 *μ*m thickness were deparaffinized and rehydrated in chloroform (3 × 10 min) and alcohol at a decreasing concentration (2 × 100%, 96%, and 70% for 2 min each) ending in distilled water. The slides were then heated in a microwave oven in 0.01 citrate buffer (pH 6.0) for 15 min for antigen retrieval. The endogenous peroxidase was blocked by a methanol/H_2_O_2_ treatment (60 mL 3% H_2_O_2_ and 190 mL methanol) for 15 min. To minimize nonspecific binding of the antibodies porcine serum (1 : 10) was applied. Tissue samples were incubated overnight at 4°C with the following primary antibodies: polyclonal goat anti-Shh (Biozol, Eching, Germany, dilution 1 : 240 in TBS), polyclonal rabbit anti-Patched (Biozol, Eching, Germany, dilution 1 : 50 in TBS), polyclonal rabbit anti-Smoothened (Biozol, Eching, Germany, dilution 1 : 200 in TBS), polyclonal rabbit anti-Gli1 (Biozol, Eching, Germany, dilution 1 : 300 in TBS), polyclonal rabbit anti-Gli2 (Abcam, Cambridge, UK, Dilution 1 : 300 in TBS), and monoclonal mouse anti-Gli3 (Biozol, Eching, Germany, clone 2C9, dilution 1 : 200 in TBS) antibodies. After specific binding of the primary antibodies corresponding biotinylated anti-mouse, anti-goat or anti-rabbit F(ab′)_2_ fragments (DAKO, Hamburg, Germany) were applied at a dilution of 1 : 400. The peroxidase-conjugated avidin-biotin complex (ABC) technique (DAKO, Hamburg, Germany) with 3,3′-diaminobenzidine (DAB, Sigma, Deisenhofen, Germany) as substrate was used to visualize the specific antigen binding (see Supplementary Figure 1 in the Supplementary Material available online at http://dx.doi.org/10.1155/2015/494269). Finally, the samples were counterstained with hematoxylin and mounted. As negative controls for each antibody, slides were incubated in TBS without primary antibody (Supplementary Figure 1). Colon carcinoma samples served as positive control for all antibodies (Supplementary Figure 1).

### 2.3. Scoring

We analyzed fetal cerebellar and telencephalic cortical layers. In the telencephalon, the marginal zone (MZ), the cortical plate (CP), the subplate (SP), the intermediate zone (IZ), the subventricular zone (SVZ), and the ventricular zone (VZ) were investigated. In the cerebellum, the external granule layer (EGL), the molecular layer (ML), and the internal granule layer (IGL) were assessed. Intensity of staining reactivity and frequency were documented by a semiquantitative score. The frequency score ranged from 0 to 4; meaning 0: 0-1%, 1: 1–10%, 2: 10–25%, 3: 25–50%, and 4: >50% of all cells showing a positive staining. Likewise, intensity was recorded in a similar semiquantitative approach as follows: 0: no staining, 1: weak staining, 2: moderate staining, 3: strong staining (raw data of the expression scores are given in Supplementary Tables 1 and 2). The two results for intensity of reactivity and expression frequency were multiplied, so that the “Multi-Score” reflects both [30]. The evaluation and photographic documentation of the immunohistochemical staining were performed using an Olympus BX50 light microscope.

### 2.4. Statistical Analyses

Ordinal scaled response variable (Multi-Score) and nominal explanatory variable (brain area) were analyzed with a contingency analysis and subsequently tested using the likelihood-ratio test. Multi-Score data is given as box plots in the figures. The upper and lower whisker extend from the ends of the box to the outermost data point that falls within the distances computed as follows: 3rd quartile + 1.5 *∗* (interquartile range) or 1st quartile − 1.5 *∗* (interquartile range). The association of the fetal age with the response variable (Multi-Score) was analyzed by ordinal logistic regression. The cumulative logistic probability plot provides an overview about what the logistic model is fitting. For each *x* value (WOG), the probability scale (in the *y* direction) is divided into probabilities for each response category (Multi-Score). The probabilities are measured as the vertical distance between the blue curves, with the total across all Y category probabilities sum to 1 (see http://www.jmp.com/support/downloads/pdf/jmp_stat_graph_guide.pdf). Subsequently, the hypothesis that the week of gestation has no effect on the response variables was analyzed using the likelihood-ratio Chi-square test. A significance level of alpha = 0.05 was chosen for all tests; therefore all values lower than 0.05 were considered as being significant. For adjustment of the *p*-values due to multiple testing we used the method of Bonferroni-Holm. Statistical analysis was performed using JMP 8.0.1 software (SAS, Cary, NC, USA).

## 3. Results

### 3.1. Strongest Expression Levels of the SHH Pathway Are Detected in the Cortical Plate and the Ventricular Zone in the Human Fetal Telencephalon

**Figure 1:**
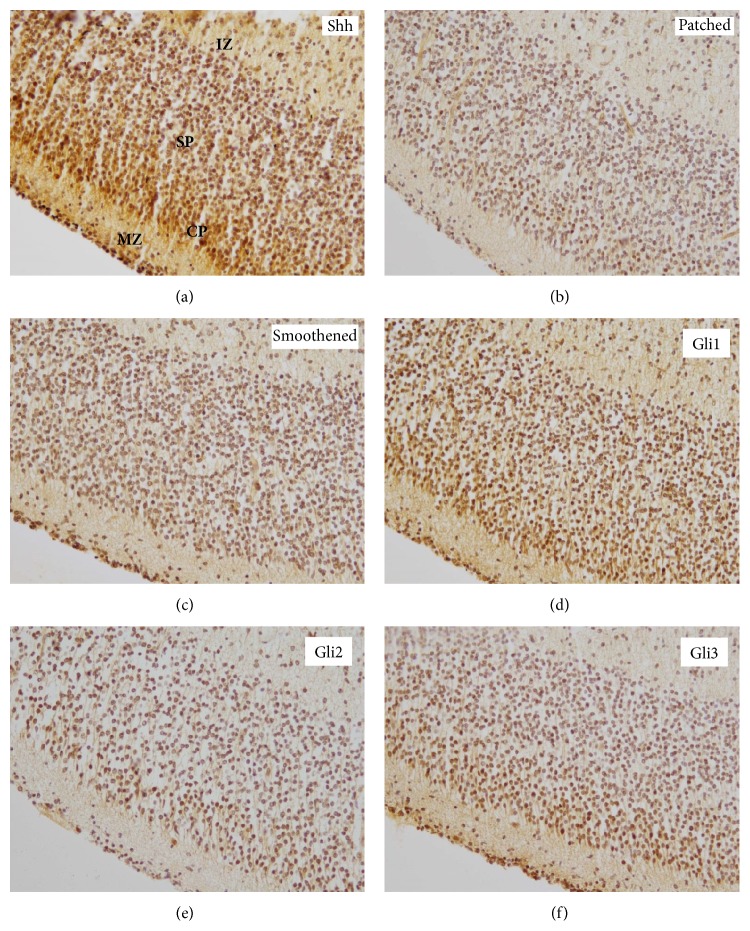
Expression pattern of* SHH* pathway members in the normal human fetal cortex. Immunohistochemistry for (a) Shh, (b) Patched, (c) Smoothened, (d) Gli1, (e) Gli2, and (f) Gli3 in superficial telencephalic cortical layers including MZ (marginal zone), CP (cortical plate), SP (subplate), and IZ (intermediate zone) (examples are depicted from a 14th week of gestation fetus; original magnification: 200x).

**Figure 2:**
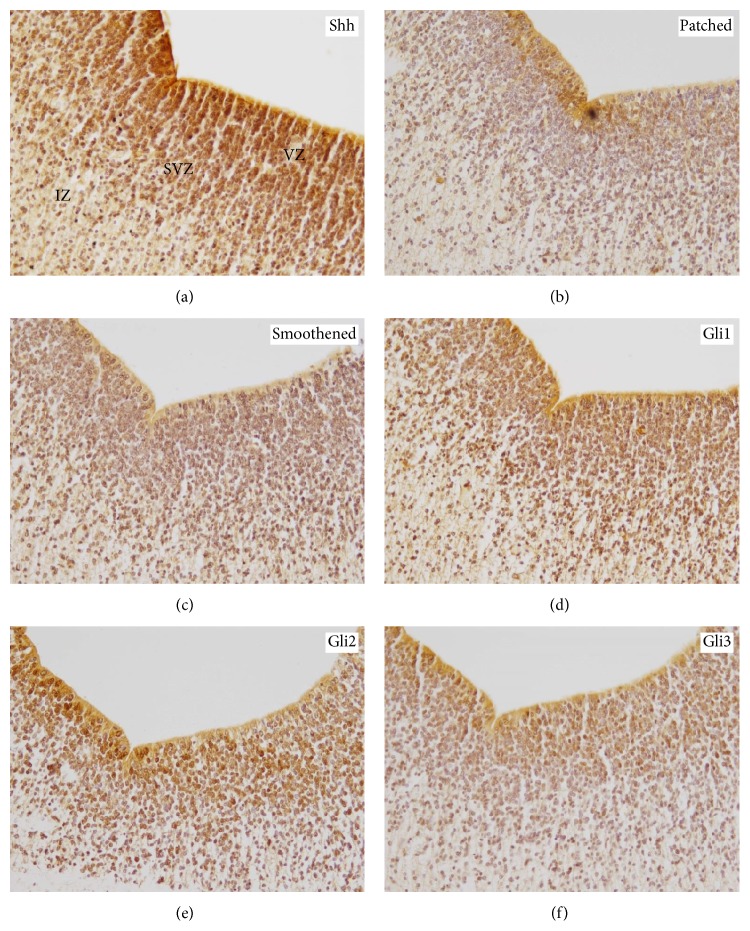
Expression pattern of* SHH* pathway members in the normal human fetal cortex. Immunohistochemistry for (a) Shh, (b) Patched, (c) Smoothened, (d) Gli1, (e) Gli2, and (f) Gli3 in the internal telencephalic layers including IZ (intermediate zone), SVZ (subventricular zone), and VZ (ventricular zone) (examples are depicted from a 14th week of gestation fetus; original magnification: 200x).

**Figure 3:**
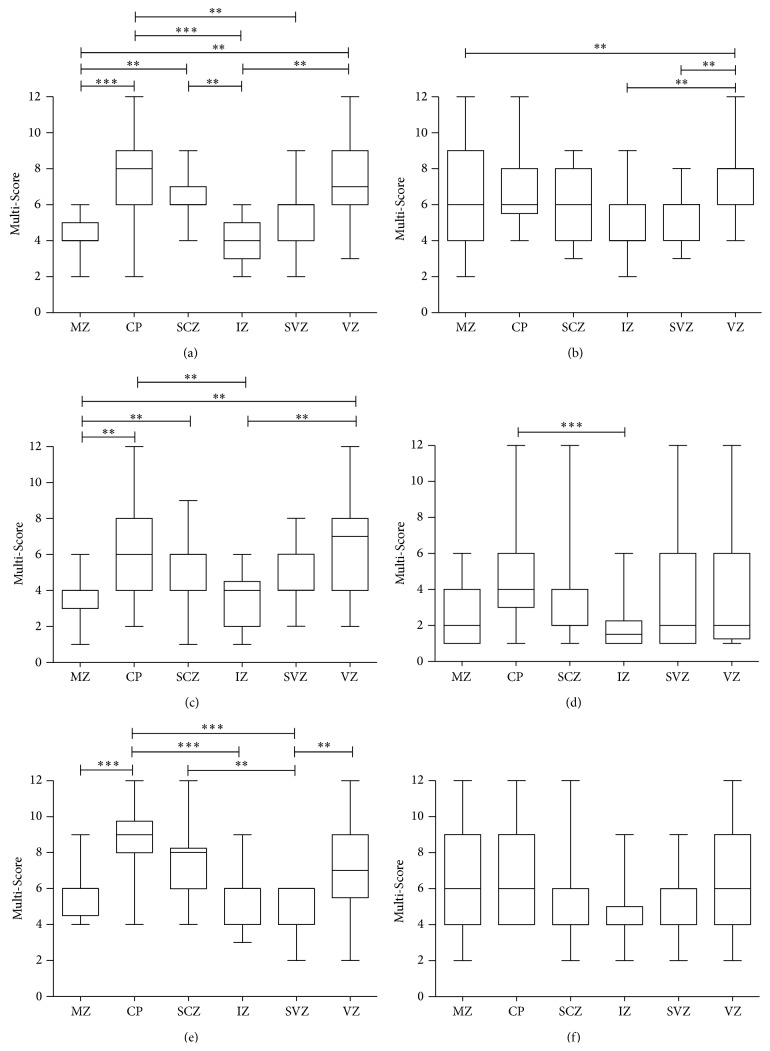
*SHH* pathway members are differentially expressed in the developing human telencephalon. Box plots of Multi-Score expression values in different telencephalic areas are depicted for (a) Shh, (b) Patched, (c) Smoothened, (d) Gli1, (e) Gli2, and (f) Gli3. For each factor *n* = 22 samples were assessed and expression values compared by a contingency analysis followed by the likelihood-ratio test (^*∗*^ *p* < 0.05; ^*∗∗*^ *p* < 0.01; ^*∗∗∗*^ *p* < 0.0001).

In the human fetal telencephalon, SHH was most strongly expressed in the CP (median: 8; range: 2–12) and the VZ (median: 7; range: 3–12), while its expression in the MZ (median: 4; range: 2–6) and the IZ (median: 4; range: 2–6) was significantly lower. Intermediate Shh levels were detected in the SP (median: 6; range: 4–9) showing significantly higher expression as compared to the MZ and IZ, however not considerably differing from the levels in the CP or VZ. The SVZ (median: 6; range: 2–9) only displayed lower Shh levels as compared to the CP (Figures 1(a), 2(a), and 3(a)). In contrast, Patched expression was homogeneously distributed over the different telencephalic layers, only showing significantly higher levels in the VZ (median: 8; range: 4–12) as compared to the MZ (median: 6; range: 2–12), the SVZ (median: 6; range: 3–8), and the IZ (median: 4; range: 2–9) (Figures 1(b), 2(b), and 3(b)). The expression pattern of Smoothened was similar to Shh, showing no significant difference between the CP (median: 6; range: 2–12) and the SVZ (median: 4; range: 2–8), however lacking the significant difference between the SP (median: 6; range: 1–9) and the IZ (median: 4; range: 1–6). The CP still showed higher levels of Smoothened as compared to the MZ (median: 4; range: 1–6) and IZ (Figures 1(c), 2(c), and 3(c)). In contrast to the Shh expression pattern, Gli1 was only very moderately regulated in the human fetal cortex; however the CP (median: 4; range: 1–12) still exhibited significantly higher levels as compared to the IZ (median: 1,5; range: 1–6) (Figures 1(d), 2(d), and 3(d)). The highest expression of Gli2 was found in the CP (median: 6; range: 4–12) and the SP (median: 8; range: 4–12). The CP showed significantly stronger Gli2 expression levels as compared to the MZ (median: 6; range: 4–9), IZ (median: 6; range: 3–9), and the SVZ (median: 6; range: 2–6). In addition, the expression of Gli2 was significantly lower in the SVZ as compared to the SP (median 8; range: 4–12) and the VZ (median: 7; range: 2–12) (Figures 1(e), 2(e), and 3(e)). Finally the analysis of Gli3 expression in the human fetal telencephalon revealed no significant differences in the distinct cortical layers (Figures 1(f), 2(f), and 3(f)). Shh was the only molecule showing a diffuse expression comprising intra- and extracellular compartments, while Patched and Smoothened exhibited a cellular localisation. The Gli proteins mostly but not exclusively displayed a nuclear staining pattern. The expression profiles of the* SHH* pathway members rather showed a gradual increase or decrease in the transition zones between distinct cortical layers than a clear-cut change in staining intensity or frequency. Since staining intensity was only indirectly reflected by the multi-scoring system, we additionally analyzed if any specific areas showed considerable differences in the staining intensity for the* SHH* pathway molecules. While the staining intensity of most* SHH* pathway members remained unchanged over the telencephalic areas, Shh itself was most intensively expressed in the CP and the VZ (Figures 1(a)–1(f) and 2(a)–2(f)). Detailed statistical comparisons are indicated in Supplementary Table 3.

### 3.2. In the Human Fetal Cerebellum Strongest Expression of the SHH Pathway Members Is Detected in the EGL and the IGL

**Figure 4:**
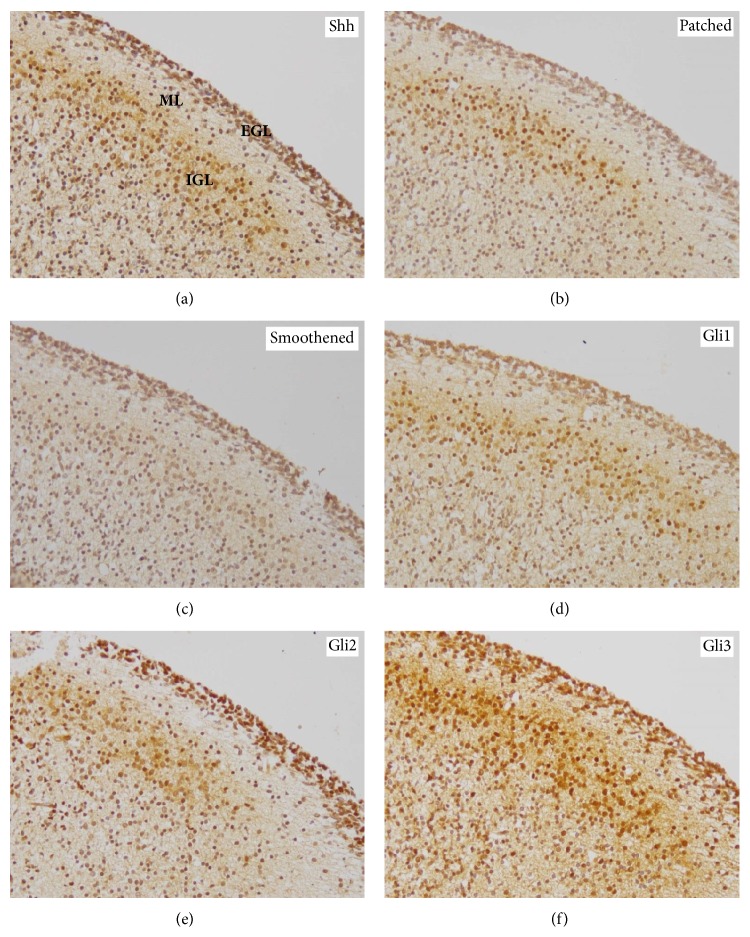
Expression patterns of* SHH* pathway members in the normal human fetal cerebellum. Immunohistochemistry for (a) Shh, (b) Patched, (c) Smoothened, (d) Gli1, (e) Gli2, and (f) Gli3 in the cerebellum including EGL (external granular layer), ML (molecular layer), and IGL (internal granular layer) (examples are depicted from a 18th week of gestation fetus; original magnification: 200x).

**Figure 5:**
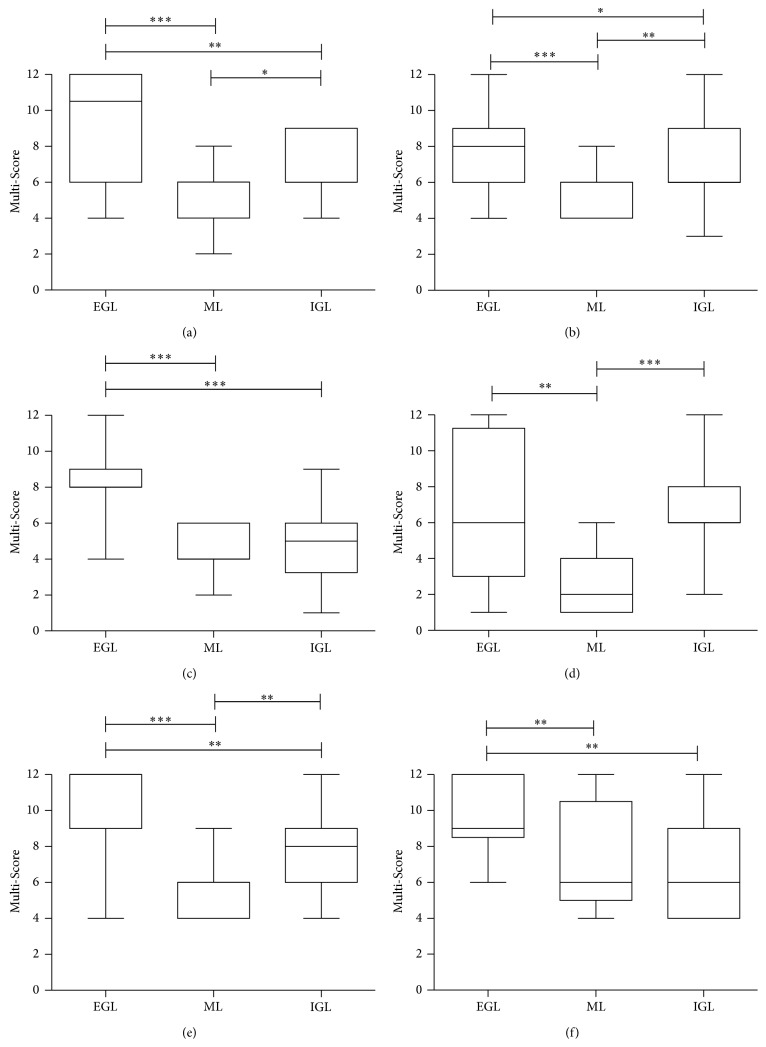
*SHH* pathway members are differentially expressed in the developing human cerebellum. Box plots of Multi-Score expression values in different cerebellar areas are depicted for (a) Shh, (b) Patched, (c) Smoothened, (d) Gli1, (e) Gli2, and (f) Gli3. For each factor *n* = 22 samples were assessed and expression values compared by a contingency analysis followed by the likelihood-ratio test (^*∗*^ *p* < 0.05; ^*∗∗*^ *p* < 0.01; ^*∗∗∗*^ *p* < 0.0001).

The members of the SHH pathway showed a similar expression pattern in human fetal cerebellar layers. Strongest* SHH* expression was found in the EGL (median: 10.5; range: 4–12), whereas its expression was significantly lower in the ML (median: 6; range: 2–8) and in the IGL (median: 6; range: 4–9). The IGL also showed significantly stronger Shh levels as compared to the ML (Figures 4(a) and 5(a)). Similarly, Patched expression in the EGL (median: 8; range: 4–12) was also significantly stronger than in the ML (median: 6; range: 4–8) and the IGL (median: 6; range: 3–12). Additionally, the expression of Patched in the IGL was significantly stronger as compared to the ML (Figures 4(b) and 5(b)). The Smoothened expression pattern was similar to Shh showing significantly stronger levels in the EGL (median: 8; range: 4–12) than in the ML (median: 4; range: 2–6) or the IGL (median: 5; range: 1–9) (Figures 4(c) and 5(c)). Gli1 was most strongly expressed in the EGL (median: 6; range: 1–12) and IGL (median: 6; range: 2–12), while its expression in the ML (median: 2; range: 2–12) was significantly lower as compared to both of them (Figures 4(d) and 5(d)). Gli2 expression was significantly stronger in the EGL (median: 12; range: 4−12) as compared to the ML (median: 6; range: 4–9) and the IGL (median: 8; range: 4–12). In addition, the ML showed significantly lower Gli2 levels as compared to the IGL (Figures 4(e) and 5(e)). Gli3 expression was significantly stronger in the EGL (median: 9; range: 6–12) as compared to the ML (median: 6; range: 4–12) and the IGL (median: 6; range: 4–12) (Figures 4(f) and 5(f)). In contrast to the findings in the telencephalic layers, a less gradual but more abrupt change in the expression of* SHH* pathway molecules was observed (Figures 4(a)–4(f)). Furthermore, no considerable changes in the staining intensity could be detected for the* SHH* pathway molecules over the fetal cerebellar layers (Figures 4(a)–4(f)). Detailed statistical comparisons are given in Supplementary Table 4.

### 3.3. Shh Pathway Expression Increases in Superficial Telencephalic Layers but Decreases in Periventricular Zones with the Week of Gestation

**Figure 6:**
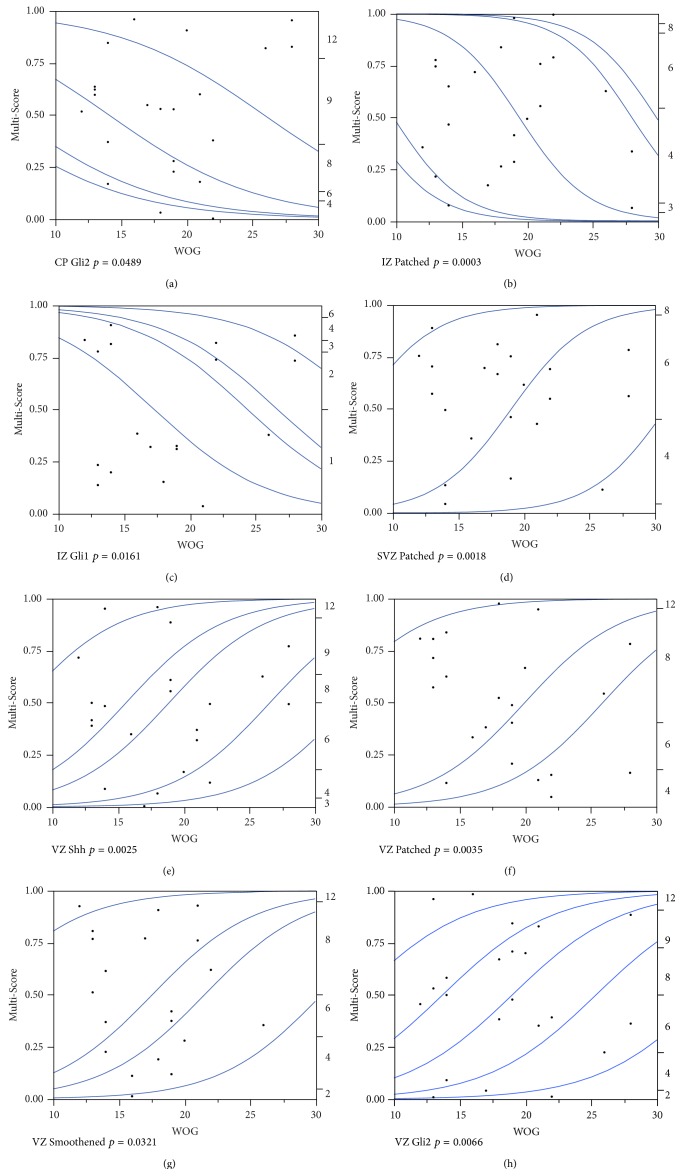
*SHH* pathway members are differentially regulated during gestation in the developing human telencephalon. (a–h) Areas showing a significant association of fetal age (week of gestation: WOG) with the response variable (Multi-Score) were analyzed by ordinal logistic regression followed by the likelihood-ratio Chi-square test. *p*-values are indicated accordingly. The cumulative logistic probability plot provides an overview about what the logistic model is fitting. For each *x* value (WOG), the probability scale (in the *y* direction) is partitioned into probabilities for each response category (Multi-Score). The probabilities are measured as the vertical distance between the blue curves, with the total across all Y category probabilities sum to 1.

While in the MZ, no significant changes were found for all molecules of the* SHH* pathway, in the CP only Gli2 expression significantly increased (*p* = 0.0489) with the week of gestation (Figure 6(a)). A significantly increasing expression was found for both Patched (*p* = 0.0003) and Gli1 (*p* = 0.0161) in the IZ (Figures 6(b) and 6(c)). In contrast to the increasing expression profiles of the Shh pathway members in the superficial telencephalic layers, their expression levels decreased over time in periventricular regions. While in the SVZ, only Patched (*p* = 0.0018) expression levels significantly decreased during pregnancy (Figure 6(d)); 4 out of the 6 investigated molecules of the Shh pathway showed a significant decreased expression in the VZ: Shh (*p* = 0.0025), Patched (*p* = 0.0035), Smoothened (*p* = 0.0321), and Gli2 (*p* = 0.0066) (Figures 6(e)–6(h)).

### 3.4. In the Human Fetal Cerebellum SHH Pathway Activation Decreases with the Week of Gestation

**Figure 7:**
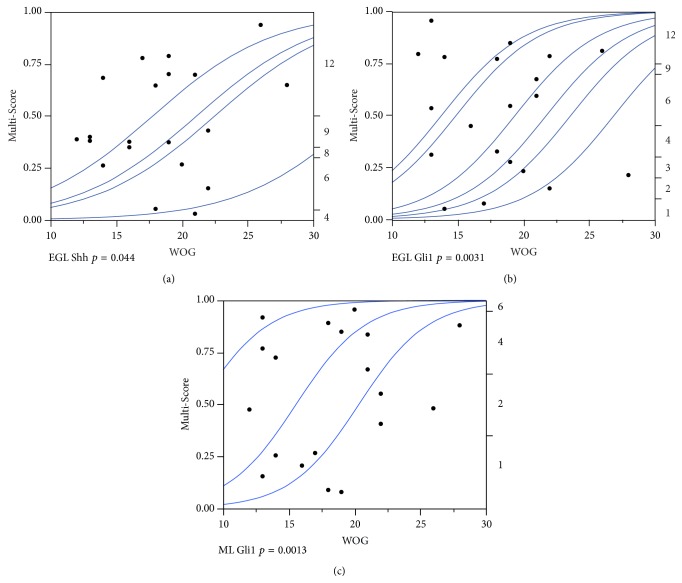
*SHH* pathway members are differentially regulated during gestation in the developing human cerebellum. (a–c) Areas showing a significant association of fetal age (week of gestation: WOG) with the response variable (Multi-Score) were analyzed by ordinal logistic regression followed by the likelihood-ratio Chi-square test. *p*-values are indicated accordingly. The cumulative logistic probability plot provides an overview about what the logistic model is fitting. For each *x* value (WOG), the probability scale (in the *y* direction) is partitioned into probabilities for each response category (Multi-Score). The probabilities are measured as the vertical distance between the blue curves, with the total across all Y category probabilities sum to 1.

**Figure 8:**
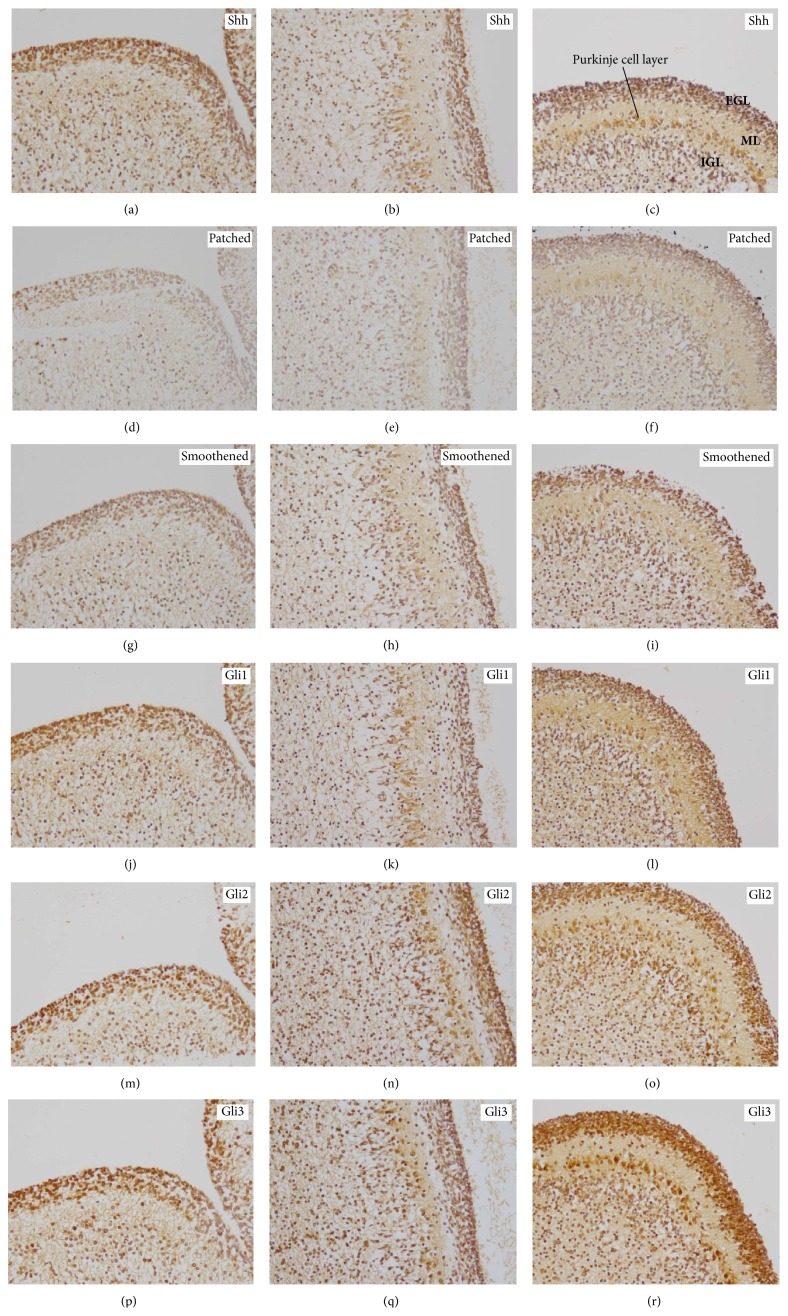
Temporal expression patterns of* SHH* pathway members in the normal human fetal cerebellum. Immunohistochemistry for (a, b, c) Shh, (d, e, f) Patched, (g, h, i) Smoothened, (j, k, l) Gli1, (m, n, o) Gli2, and (p, q, r) Gli3 showing examples of the cerebellar layers including EGL (external granular layer), ML (molecular layer), and IGL (internal granular layer). (a, d, g, j, m, p) 12th week of gestation, (b, e, h, k, n, q) 19th week of gestation, and (c, f, i, l, o, r) 28th week of gestation; original magnification: 200x.

In the EGL of the human fetal cerebellum, a significant decrease in both Shh (*p* = 0.044) and Gli1 (*p* = 0.0031) expression was seen over time while the other molecules remained unchanged (Figures 7(a) and 7(b)). In the ML, only Gli1 (*p* = 0.0013) was significantly downregulated over time whereas all other factors displayed a stable expression (Figure 7(c)). In the IGL, no considerable expression changes were observed over time. In later stages of the fetal cerebellar development, especially, differentiating Purkinje cells exhibited an increased expression of most* SHH* pathway molecules while in other layers such as the EGL Shh and Gli1 were decreased (Figure 8).

## 4. Discussion

### 4.1. The Potential Role of the SHH Pathway in the Control of Progenitor Cell Survival and Neuronal Differentiation in the Developing Human Fetal Telencephalon

The* Hh* signaling pathway is conserved among different species and plays a major role in the development of various organs and cell types [1]. In our study, we provide evidence that SHH pathway members are expressed in a specific manner in the different layers of the human telencephalon. In particular, Shh, Smoothened, and Gli2 showed increasing levels from MZ to CP and decreasing levels from SP to IZ. Strongest expression of these factors was seen in the CP and VZ. Patched also showed high expression levels in MZ, CP, and VZ but low expression in the IZ and SVZ. Concerning the temporal expression pattern, Shh, Smoothened, Gli2, and Patched levels decreased in the VZ with the week of gestation. In contrast, in the CP Gli2 is the only molecule of the* SHH* signaling pathway which increased during pregnancy.

One of the first steps in CNS development is the proliferation of precursor cells and the differentiation of neuronal progenitors into subclasses of mature neurons [31]. In the zebrafish, Shh is essential for the development of the time-dependent formation of the optical tectum [32]. In addition, in higher vertebrates, Shh regulates the proliferation of neural precursor cells, especially in the developing VZ/SVZ [33, 34]. This goes along with our findings of high expression levels of the* SHH* pathway members, especially in the VZ/SVZ. The findings that highest expression levels of Shh and its pathway members were found in the VZ and decreased with the week of gestation might be related to the high proliferative capacity of this brain area at very early stages of CNS development. Further evidence for the important role of a fine-tuned* SHH* pathway activation in progenitor cell cycle regulation is provided by findings in transgenic, Shh-overexpressing mice that develop hyperplasia of the spinal cord and the VZ during embryogenesis [34]. On the other hand, Palma et al. showed that a selective inhibition of Shh leads to a significant decrease in the size of the murine SVZ [35]. In contrast, a downregulation of the Shh signaling is important to slow down cell division and further allows the cells to exit the cell cycle which is necessary for the transition to nondividing neurons migrating away from the proliferation zones [36]. The hypothesis that murine radial glial cells (RGC), intermediate progenitor cells (IPC), and mature neurons in the SVZ need low Shh levels to regulate their generation, proliferation, and cellular migration is also corroborated by our locoregional distribution pattern in the human fetal brain showing strongest downregulation of the* SHH* pathway between the SVZ and the IZ [36].

### 4.2. Expression of Gli Family Members in the Fetal Telencephalon Is Associated with Areas Involved in Proliferation and Differentiation

The pattern of Gli1 and Gli2 expression is strikingly similar to other data concerning murine telencephalon development where Gli1 expression is induced by Shh and therefore also detected in close proximity to the Shh expression signal [13]. However, previous studies with Gli1−/− mouse mutants revealed no detectable deficits in cortical development [37]. This leads to the assumption that there is no isolated function of Gli1 regarding proliferation or migration. In contrast, Gli2−/− mouse mutants demonstrated a decrease in proliferation mainly in, otherwise, highly proliferative cortical layers [38]. This data indicates that Gli2 might be a more important activating factor in the* SHH* pathway cascade which goes well along with our findings of almost identical expression pattern for Shh and Gli2. Although the expression profile of Gli3 moderately resembles the Gli1 and Gli2 signature, no significant differences between the developing telencephalic layers were observed. However, it remains to be determined if Gli3 exerts a more inhibitory or activating function during human fetal brain development [39]. Different studies showed a correlation of Gli3 activation and cell proliferation resulting in significantly smaller neocortical layers in* Gli3*−/− mice [38, 40]. Interestingly,* Gli3/SHH* double mutants displayed a rescue of the brain malformation caused by* SHH* single mutants [24, 41]. In addition many severe malformations occurring in Shh single mutants are restored in embryos simultaneously lacking Gli3 and Shh function [22]. Therefore, Shh may modulate the activating and inhibitory function of Gli3, so that, in the absence of Shh, Gli3 will be processed to the repressor form. This indicates that Gli3 alone or the balances of both proteins are required for the normal fetal brain development in the telencephalon [42].

### 4.3. The Temporal Expression Pattern of the SHH Pathway in the Human Fetal Telencephalon Indicates a Dichotomous Role in Proliferation and Migration of Progenitor Cells

In the normal developing human fetal brain neuronal precursor cells migrate radially away from the proliferation zones (VZ/SVZ) to the surface of the brain leading to the formation of the typical six-layered neocortex [43]. Therefore migratory processes in the developing fetal telencephalon are closely associated with cell cycle exit of neuronal progenitor cells [44]. In contrast to the decrease of the proliferative activity in the VZ/SVZ, the proliferation in the IZ and the SP increases until the end of the last pregnancy trimester [45]. Accordingly, we found increasing expression levels of Patched and Gli1 in the IZ with the week of gestation. While strong expression levels of all* SHH* pathway members were observed in the CP, only Gli2 increased significantly with the week of gestation. One possible explanation for the constantly high expression levels of the* SHH* pathway members in the CP could be that the proliferation activity already starts in the 8th week of gestation while our study could only investigate human fetal brains deriving from the 12th until the 28th week of gestation. It is well known that neocortical migration and laminar organization not only depends on radial migration. Angot et al. provided evidence that Shh is a chemoattractive molecule acting on neuronal progenitors in the adult SVZ which in turn change their migratory behaviour towards an ectopic Shh expression [46]. This might be related to our findings showing constantly high expression levels of the* SHH* pathway members in the CP but decreasing levels in VZ/SVZ over time. Komada and colleagues showed that in wild-type mice the early-born neurons form the deeper layer and the late-born neurons are the source for the superficial layers of the CP. In contrast, in* SMO* knockout mice lacking normal Shh signalling, the early-born neurons were diffusely spread over the dorsal telencephalon and the late-born did not even migrate to the CP [36]. Again, this illustrates the importance of a coordinate temporal regulation of the cell cycle and a successful migration/lamination process both depending on a well-functioning* SHH* pathway.

### 4.4. Temporospatial Changes in the Sources of Shh Expression in Human Cerebellar Development Are Related to Proliferation, Migration, and Differentiation Processes

The human cerebellum consists of a three-layered structure of which the outermost, external granular layer (EGL) is formed by cerebellar precursor cells invading from the rhombic lip and then leading to the formation of the proliferating granule cells of the expanding EGL [47]. After proliferation these cells exit the cell cycle and migrate along the Bergman glia to deeper layers where they form the inner granular layer (IGL) [21, 47, 48]. The formation of the IGL lasts until several months after birth in the human brain [49]. The* SHH* pathway is supposed to play a major role not only in proliferation of the granule cells but also in other precursor cell types in the developing CNS [48]. In mice, the blocking of the Shh signaling results in a reduced proliferation of the granule precursors cells in the EGL in vivo [48] and in vitro [21]. These findings indicate that Shh secreted by EGL or Purkinje neurons is necessary for the proliferation and migration of the granule precursor cells [50]. The high Shh levels in the EGL might confirm the hypothesis that Shh plays a role as enhancer of proliferation of granule precursor cells, indicating a similar activity of Shh in the human and murine fetal cerebellum [21, 51]. Beside the expression of Shh in the human EGL, we also detected high Shh levels in the IGL while being considerably lower in the ML. To stop cell division and allow an exit of the cell cycle, for example, to migrate, a lowering of the Shh signaling is required [36]. With regard to the temporal distribution pattern, Shh significantly decreased between the 12th and 28th week of gestation in the EGL potentially reflecting a reduction in cellular proliferation and migration to the IGL. Our results confirm recently published data showing a strong Shh expression in the EGL between the 10th and 20th week of gestation [52]. In our cohort Shh is still present in the IGL until the 28th week of gestation and is not considerably downregulated if compared to the EGL. This could be due to the fact that Shh downregulation might take place at the time of birth or soon after in humans [52]. On the other hand it is known that Shh plays a role in cell differentiation which is necessary in the IGL after the immigration of granule cells [53]. Shh is further present in the human EGL until week 17 and decreases after the 20th week when Purkinje neurons form a monolayer suggesting the Purkinje neurons as the source of the Shh signaling [52].

Additionally, our investigation of the other members of the* SHH* pathway in the developing cerebellum revealed strongest expression levels in the EGL and IGL suggesting a* SHH* pathway activation in these respective cerebellar areas. In particular, the expression of Gli1, which can be transcriptionally activated by* SHH* [13] and therefore be used as a surrogate parameter for Shh activity [53], decreases in the EGL with the week of gestation indicating an inhibition of the Shh pathway, a finding which was also seen in mouse embryos [21]. In contrast to Patched, Smoothened, Gli2, or Gli3, Gli1 also showed lower expression levels in the ML over the time of pregnancy. The ongoing expression of* SHH* pathway members in other layers compared to the EGL suggested that there has to be another source for the Shh expression in the developing cerebellum. Purkinje cells seem to be a potential cell type because these cells require intact Shh signaling for proper development and maturation, a process that lasts for several months after birth [21]. We could also confirm that especially differentiating Purkinje cells show an increased expression of Shh also in later stages of the human fetal cerebellar development while other layers such as the EGL showed decreased Shh levels. Concerning CNS tumors, the medulloblastoma represents the most frequent malignant brain tumor of the childhood and is currently classified at a molecular level indicating Shh pathway disturbances as one of the key players for tumor development [54]. Additionally, it is predicted that the source of medulloblastoma subtypes is the granule neural precursor cells in the EGL being unable to exit the cell cycle due to overactivated Shh signaling [55].

## 5. Conclusion

In our study we show that the* SHH* pathway is active during different stages of the normal development in the different layers of the human brain. Thus we could provide evidence that a fine-tuned temporal Shh signaling is necessary for normal human CNS development.

## Supplementary Material

Supplementary Figure 1: Immunohistochemical staining controls. First column depicts the specific antibody staining results for each antibody used in the study (original magnification: 100x). Second column represents higher magnification images of the area indicated with the black rectagle in the first column (original magnification: 400x). The third column displays the identical specimens as shown in the first and second column without antibody staining serving as negative controls (original magnification: 400x). The forth column shows colon carcinoma samples serving as positive control for each antibody (original magnification).
Supplementary Table 1: Protein expression raw data for staining frequency, staining intensity and the resulting multi-score in the telencephalic layers (empty boxes: data acquisition was not possible due to missing tissue specimens or other technical reasons).
Supplementary Table 2: Protein expression raw data for staining frequency, staining intensity and the resulting multi-score in the cerebellar layers (empty boxes: data acquisition was not possible due to missing tissue specimens or other technical reasons).
Supplementary Table 3: Comparison of multi-scores/region in the telencephalon assessed by likelihood-ratio test (∗not significant after Bonferroni-Holm correction).
Supplementary Table 4: Comparison of multi-scores/region in the cerebellum assessed by likelihood-ratio test (∗not significant after Bonferroni-Holm correction).

## Figures and Tables

**Table 1:** Clinical and epidemiological data.

Case	Week of gestation	Clinical data
1	20	Induced abortion, cardiac malformation
2	16	Intrauterine death, collapsed amniotic sac
3	14	Induced abortion, preterm amniorrhexis
4	12	Intrauterine death, ruptured gravidity
5	22	Postpartal death, preterm birth
6	13	Ejection
7	14	Intrauterine death, preterm amniorrhexis
8	18	Intrauterine death, preterm amniorrhexis
9	22	Intrauterine death, preterm amniorrhexis
10	21	Intrauterine death, anhydramnion
11	28	Intrauterine death, twin-to-twin transfusion syndrome
12	16	Induced abortion, suspected nephron agenesis
13	14	Induced abortion, on parents demand
14	13	Intrauterine death, preterm abruption of placenta
15	17	Intrauterine death, preterm abruption of placenta
16	19	Induced abortion, anhydramnios, gemini gravidity
17	19	Induced abortion, anhydramnios, gemini gravidity
18	21	Intrauterine death, chorioamnionitis, placentitis
19	19	Intrauterine death, chorioamnionitis, placentitis
20	18	Intrauterine death, chorioamnionitis, placentitis
21	13	Intrauterine death, chorioamnionitis, placentitis
22	26	Postpartal death, preterm abruption of placenta
