# Supplementary material for: Expression Profile of Sonic Hedgehog Pathway Members in the Developing Human Fetal Brain
Source: Biomed Res Int. 2015 Jul 21;2015:494269. doi: 10.1155/2015/494269 (PMC4523658; doi:10.1155/2015/494269)
Supplement: Supplementary file 1 — Supplementary Figure 1: Immunohistochemical staining controls. First column depicts the specific antibody staining results for each antibody used in the study (original magnification: 100x). Second column represents higher magnification images of the area indicated with the black rectagle in the first column (original magnification: 400x). The third column displays the identical specimens as shown in the first and second column without antibody staining serving as negative controls (original magnification: 400x). The forth column shows colon carcinoma samples serving as positive control for each antibody (original magnification). Supplementary Table 1: Protein expression raw data for staining frequency, staining intensity and the resulting multi-score in the telencephalic layers (empty boxes: data acquisition was not possible due to missing tissue specimens or other technical reasons). Supplementary Table 2: Protein expression raw data for staining frequency, staining intensity and the resulting multi-score in the cerebellar layers (empty boxes: data acquisition was not possible due to missing tissue specimens or other technical reasons). Supplementary Table 3: Comparison of multi-scores/region in the telencephalon assessed by likelihood-ratio test (∗not significant after Bonferroni-Holm correction). Supplementary Table 4: Comparison of multi-scores/region in the cerebellum assessed by likelihood-ratio test (∗not significant after Bonferroni-Holm correction). [file 494269.f1.pdf]

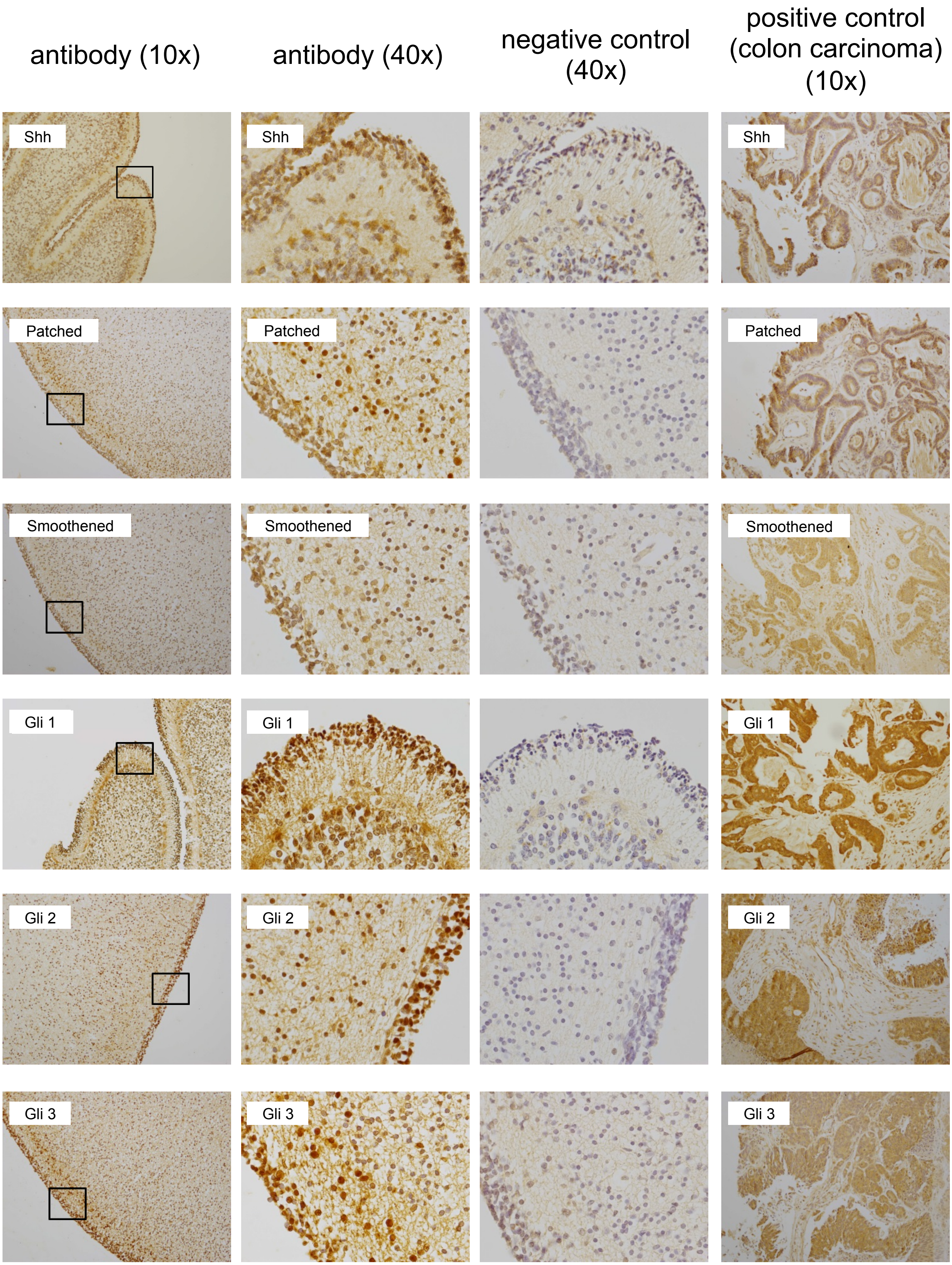
antibody (10x)
antibody (40x)
negative control (40x)
positive control (colon carcinoma) (10x)
Shh
Shh
Shh
Patched
Patched
Patched
Smoothened
Smoothened
Smoothened
Gli 1
Gli 1
Gli 1
Gli 2
Gli 2
Gli 2
Gli 3
Gli 3
Gli 3

Supplementary Table 1: Protein expression raw data for staining frequency, staining intensity and the resulting multi-score in the telencephalic layers (empty boxes: data acquisition was not possible due to missing tissue specimens or other technical reasons).

Factor	Case-ID	Week of gestation	Area	Frequency	Intensity	Multi-Score
Gli-1	ES 49/04	20	Marginal zone			
Gli-1	ES 49/04	20	Cortical plate			
Gli-1	ES 49/04	20	Subplate			
Gli-1	ES 49/04	20	Intermediate zone			
Gli-1	ES 49/04	20	Subventricular zone			
Gli-1	ES 49/04	20	Ventricular zone			
Gli-1	ES 15/04	16	Marginal zone			
Gli-1	ES 15/04	16	Cortical plate	4	2	8
Gli-1	ES 15/04	16	Subplate	2	2	4
Gli-1	ES 15/04	16	Intermediate zone	1	1	1
Gli-1	ES 15/04	16	Subventricular zone	3	2	6
Gli-1	ES 15/04	16	Ventricular zone	4	2	8
Gli-1	ES 3/04	14	Marginal zone	2	2	4
Gli-1	ES 3/04	14	Cortical plate	4	2	8
Gli-1	ES 3/04	14	Subplate	2	1	2
Gli-1	ES 3/04	14	Intermediate zone	1	1	1
Gli-1	ES 3/04	14	Subventricular zone	4	3	12
Gli-1	ES 3/04	14	Ventricular zone	4	3	12
Gli-1	ES 155/03	12	Marginal zone	3	2	6
Gli-1	ES 155/03	12	Cortical plate	4	3	12
Gli-1	ES 155/03	12	Subplate	2	2	4
Gli-1	ES 155/03	12	Intermediate zone	2	1	2
Gli-1	ES 155/03	12	Subventricular zone	3	2	6

Gli-1	ES 155/03	12	Ventricular zone	2	2	4
Gli-1	ES 148/03	22	Marginal zone	2	2	4
Gli-1	ES 148/03	22	Cortical plate	4	3	12
Gli-1	ES 148/03	22	Subplate	4	3	12
Gli-1	ES 148/03	22	Intermediate zone	3	1	3
Gli-1	ES 148/03	22	Subventricular zone	3	2	6
Gli-1	ES 148/03	22	Ventricular zone	3	2	6
Gli-1	ES 144/03	13	Marginal zone	1	2	2
Gli-1	ES 144/03	13	Cortical plate	3	2	6
Gli-1	ES 144/03	13	Subplate	1	2	2
Gli-1	ES 144/03	13	Intermediate zone	1	2	2
Gli-1	ES 144/03	13	Subventricular zone	2	1	2
Gli-1	ES 144/03	13	Ventricular zone	1	1	1
Gli-1	ES 142/03	14	Marginal zone	1	3	3
Gli-1	ES 142/03	14	Cortical plate	2	2	4
Gli-1	ES 142/03	14	Subplate	2	2	4
Gli-1	ES 142/03	14	Intermediate zone	1	2	2
Gli-1	ES 142/03	14	Subventricular zone	2	2	4
Gli-1	ES 142/03	14	Ventricular zone	1	1	1
Gli-1	ES 128/03	18	Marginal zone	1	1	1
Gli-1	ES 128/03	18	Cortical plate	3	2	6
Gli-1	ES 128/03	18	Subplate	2	2	4
Gli-1	ES 128/03	18	Intermediate zone	1	1	1
Gli-1	ES 128/03	18	Subventricular zone	2	1	2
Gli-1	ES 128/03	18	Ventricular zone	1	1	1
Gli-1	ES 118/03	22	Marginal zone	1	1	1
Gli-1	ES 118/03	22	Cortical plate	2	1	2
Gli-1	ES 118/03	22	Subplate	3	2	6
Gli-1	ES 118/03	22	Intermediate zone	2	2	4

Gli-1	ES 118/03	22	Subventricular zone			
Gli-1	ES 118/03	22	Ventricular zone			
Gli-1	ES 112/03	21	Marginal zone	1	1	1
Gli-1	ES 112/03	21	Cortical plate	2	1	2
Gli-1	ES 112/03	21	Subplate	4	2	8
Gli-1	ES 112/03	21	Intermediate zone		0	
Gli-1	ES 112/03	21	Subventricular zone	1	1	1
Gli-1	ES 112/03	21	Ventricular zone	1	1	1
Gli-1	ES 67/03	28	Marginal zone	2	2	4
Gli-1	ES 67/03	28	Cortical plate	3	2	6
Gli-1	ES 67/03	28	Subplate	2	1	2
Gli-1	ES 67/03	28	Intermediate zone	3	2	6
Gli-1	ES 67/03	28	Subventricular zone	3	2	6
Gli-1	ES 67/03	28	Ventricular zone	3	2	6
Gli-1	ES 67/03	28	Marginal zone	1	2	2
Gli-1	ES 67/03	28	Cortical plate	2	2	4
Gli-1	ES 67/03	28	Subplate	2	2	4
Gli-1	ES 67/03	28	Intermediate zone	2	2	4
Gli-1	ES 67/03	28	Subventricular zone	1	1	1
Gli-1	ES 67/03	28	Ventricular zone	2	1	2
Gli-1	ES 58/03	16	Marginal zone			
Gli-1	ES 58/03	16	Cortical plate			
Gli-1	ES 58/03	16	Subplate			
Gli-1	ES 58/03	16	Intermediate zone			
Gli-1	ES 58/03	16	Subventricular zone			
Gli-1	ES 58/03	16	Ventricular zone			
Gli-1	ES 187/02	14	Marginal zone	2	2	4
Gli-1	ES 187/02	14	Cortical plate	3	2	6
Gli-1	ES 187/02	14	Subplate	2	1	2

Gli-1	ES 187/02	14	Intermediate zone	2	1	2
Gli-1	ES 187/02	14	Subventricular zone	1	1	1
Gli-1	ES 187/02	14	Ventricular zone	1	2	2
Gli-1	ES 102/02	13	Marginal zone	1	1	1
Gli-1	ES 102/02	13	Cortical plate	2	2	4
Gli-1	ES 102/02	13	Subplate	2	1	2
Gli-1	ES 102/02	13	Intermediate zone	1	1	1
Gli-1	ES 102/02	13	Subventricular zone	2	1	2
Gli-1	ES 102/02	13	Ventricular zone	2	1	2
Gli-1	ES 38/02	17	Marginal zone	1	1	1
Gli-1	ES 38/02	17	Cortical plate	2	2	4
Gli-1	ES 38/02	17	Subplate	2	1	2
Gli-1	ES 38/02	17	Intermediate zone	1	1	1
Gli-1	ES 38/02	17	Subventricular zone	1	1	1
Gli-1	ES 38/02	17	Ventricular zone	2	1	2
Gli-1	ES 23/02	19	Marginal zone	2	1	2
Gli-1	ES 23/02	19	Cortical plate	3	2	6
Gli-1	ES 23/02	19	Subplate	4	2	8
Gli-1	ES 23/02	19	Intermediate zone	1	1	1
Gli-1	ES 23/02	19	Subventricular zone	3	2	6
Gli-1	ES 23/02	19	Ventricular zone	3	2	6
Gli-1	ES 22/02	19	Marginal zone	1	1	1
Gli-1	ES 22/02	19	Cortical plate	3	2	6
Gli-1	ES 22/02	19	Subplate	2	2	4
Gli-1	ES 22/02	19	Intermediate zone	1	1	1
Gli-1	ES 22/02	19	Subventricular zone	2	2	4
Gli-1	ES 22/02	19	Ventricular zone	3	2	6
Gli-1	ES 15/02	21	Marginal zone	1	1	1
Gli-1	ES 15/02	21	Cortical plate	2	1	2

Gli-1	ES 15/02	21	Subplate	2	1	2
Gli-1	ES 15/02	21	Intermediate zone	1	1	1
Gli-1	ES 15/02	21	Subventricular zone	1	1	1
Gli-1	ES 15/02	21	Ventricular zone	2	1	2
Gli-1	ES 4/02	19	Marginal zone	1	2	2
Gli-1	ES 4/02	19	Cortical plate	1	1	1
Gli-1	ES 4/02	19	Subplate	1	1	1
Gli-1	ES 4/02	19	Intermediate zone		0	
Gli-1	ES 4/02	19	Subventricular zone	1	1	1
Gli-1	ES 4/02	19	Ventricular zone	1	1	1
Gli-1	ES 154/01	18	Marginal zone	1	2	2
Gli-1	ES 154/01	18	Cortical plate	2	1	2
Gli-1	ES 154/01	18	Subplate	1	1	1
Gli-1	ES 154/01	18	Intermediate zone		0	
Gli-1	ES 154/01	18	Subventricular zone	2	1	2
Gli-1	ES 154/01	18	Ventricular zone	2	1	2
Gli-1	ES 151/01	13	Marginal zone	2	2	4
Gli-1	ES 151/01	13	Cortical plate	2	2	4
Gli-1	ES 151/01	13	Subplate	2	1	2
Gli-1	ES 151/01	13	Intermediate zone	1	1	1
Gli-1	ES 151/01	13	Subventricular zone	2	1	2
Gli-1	ES 151/01	13	Ventricular zone	2	2	4
Gli-1	ES 129/01	26	Marginal zone	1	2	2
Gli-1	ES 129/01	26	Cortical plate	2	2	4
Gli-1	ES 129/01	26	Subplate	1	1	1
Gli-1	ES 129/01	26	Intermediate zone	1	2	2
Gli-1	ES 129/01	26	Subventricular zone	1	1	1
Gli-1	ES 129/01	26	Ventricular zone	2	1	2
Gli-2	ES 49/04	20	Marginal zone	2	3	6

Gli-2	ES 49/04	20	Cortical plate	4	3	12
Gli-2	ES 49/04	20	Subplate	3	3	9
Gli-2	ES 49/04	20	Intermediate zone	3	3	9
Gli-2	ES 49/04	20	Subventricular zone	3	2	6
Gli-2	ES 49/04	20	Ventricular zone	4	2	8
Gli-2	ES 15/04	16	Marginal zone	2	3	6
Gli-2	ES 15/04	16	Cortical plate	4	3	12
Gli-2	ES 15/04	16	Subplate	4	2	8
Gli-2	ES 15/04	16	Intermediate zone	2	2	4
Gli-2	ES 15/04	16	Subventricular zone	2	3	6
Gli-2	ES 15/04	16	Ventricular zone	4	3	12
Gli-2	ES 3/04	14	Marginal zone	2	3	6
Gli-2	ES 3/04	14	Cortical plate	2	3	6
Gli-2	ES 3/04	14	Subplate	3	3	9
Gli-2	ES 3/04	14	Intermediate zone	2	2	4
Gli-2	ES 3/04	14	Subventricular zone	2	2	4
Gli-2	ES 3/04	14	Ventricular zone	3	3	9
Gli-2	ES 155/03	12	Marginal zone			
Gli-2	ES 155/03	12	Cortical plate	4	2	8
Gli-2	ES 155/03	12	Subplate	4	2	8
Gli-2	ES 155/03	12	Intermediate zone	2	2	4
Gli-2	ES 155/03	12	Subventricular zone	3	2	6
Gli-2	ES 155/03	12	Ventricular zone	3	3	9
Gli-2	ES 148/03	22	Marginal zone	2	3	6
Gli-2	ES 148/03	22	Cortical plate	3	3	9
Gli-2	ES 148/03	22	Subplate	2	3	6
Gli-2	ES 148/03	22	Intermediate zone	1	3	3
Gli-2	ES 148/03	22	Subventricular zone	2	2	4
Gli-2	ES 148/03	22	Ventricular zone	2	3	6

Gli-2	ES 144/03	13	Marginal zone	3	3	9
Gli-2	ES 144/03	13	Cortical plate	3	3	9
Gli-2	ES 144/03	13	Subplate	3	2	6
Gli-2	ES 144/03	13	Intermediate zone	3	2	6
Gli-2	ES 144/03	13	Subventricular zone	2	2	4
Gli-2	ES 144/03	13	Ventricular zone	3	3	9
Gli-2	ES 142/03	14	Marginal zone	2	2	4
Gli-2	ES 142/03	14	Cortical plate	4	2	8
Gli-2	ES 142/03	14	Subplate	3	3	9
Gli-2	ES 142/03	14	Intermediate zone	3	2	6
Gli-2	ES 142/03	14	Subventricular zone	2	2	4
Gli-2	ES 142/03	14	Ventricular zone	2	3	6
Gli-2	ES 128/03	18	Marginal zone	2	2	4
Gli-2	ES 128/03	18	Cortical plate	3	3	9
Gli-2	ES 128/03	18	Subplate	2	3	6
Gli-2	ES 128/03	18	Intermediate zone	2	3	6
Gli-2	ES 128/03	18	Subventricular zone	2	2	4
Gli-2	ES 128/03	18	Ventricular zone	3	2	6
Gli-2	ES 118/03	22	Marginal zone	2	3	6
Gli-2	ES 118/03	22	Cortical plate	2	2	4
Gli-2	ES 118/03	22	Subplate	3	2	6
Gli-2	ES 118/03	22	Intermediate zone	2	3	6
Gli-2	ES 118/03	22	Subventricular zone	1	2	2
Gli-2	ES 118/03	22	Ventricular zone	2	1	2
Gli-2	ES 112/03	21	Marginal zone	3	3	9
Gli-2	ES 112/03	21	Cortical plate	3	3	9
Gli-2	ES 112/03	21	Subplate	4	3	12
Gli-2	ES 112/03	21	Intermediate zone	2	3	6
Gli-2	ES 112/03	21	Subventricular zone	2	3	6

Gli-2	ES 112/03	21	Ventricular zone	2	3	6
Gli-2	ES 67/03	28	Marginal zone	2	3	6
Gli-2	ES 67/03	28	Cortical plate	4	3	12
Gli-2	ES 67/03	28	Subplate	4	2	8
Gli-2	ES 67/03	28	Intermediate zone	2	3	6
Gli-2	ES 67/03	28	Subventricular zone	3	2	6
Gli-2	ES 67/03	28	Ventricular zone	2	3	6
Gli-2	ES 67/03	28	Marginal zone	2	3	6
Gli-2	ES 67/03	28	Cortical plate	4	3	12
Gli-2	ES 67/03	28	Subplate	4	2	8
Gli-2	ES 67/03	28	Intermediate zone	3	3	9
Gli-2	ES 67/03	28	Subventricular zone	3	2	6
Gli-2	ES 67/03	28	Ventricular zone	2	2	4
Gli-2	ES 58/03	16	Marginal zone			
Gli-2	ES 58/03	16	Cortical plate			
Gli-2	ES 58/03	16	Subplate			
Gli-2	ES 58/03	16	Intermediate zone			
Gli-2	ES 58/03	16	Subventricular zone			
Gli-2	ES 58/03	16	Ventricular zone			
Gli-2	ES 187/02	14	Marginal zone	2	3	6
Gli-2	ES 187/02	14	Cortical plate	3	3	9
Gli-2	ES 187/02	14	Subplate	2	2	4
Gli-2	ES 187/02	14	Intermediate zone	2	3	6
Gli-2	ES 187/02	14	Subventricular zone	3	2	6
Gli-2	ES 187/02	14	Ventricular zone	4	2	8
Gli-2	ES 102/02	13	Marginal zone	3	3	9
Gli-2	ES 102/02	13	Cortical plate	3	3	9
Gli-2	ES 102/02	13	Subplate	4	2	8
Gli-2	ES 102/02	13	Intermediate zone	2	2	4

Gli-2	ES 102/02	13	Subventricular zone	2	2	4
Gli-2	ES 102/02	13	Ventricular zone	2	2	4
Gli-2	ES 38/02	17	Marginal zone	2	2	4
Gli-2	ES 38/02	17	Cortical plate	3	3	9
Gli-2	ES 38/02	17	Subplate	2	2	4
Gli-2	ES 38/02	17	Intermediate zone	3	2	6
Gli-2	ES 38/02	17	Subventricular zone	2	2	4
Gli-2	ES 38/02	17	Ventricular zone	2	2	4
Gli-2	ES 23/02	19	Marginal zone	2	2	4
Gli-2	ES 23/02	19	Cortical plate	3	3	9
Gli-2	ES 23/02	19	Subplate	3	2	6
Gli-2	ES 23/02	19	Intermediate zone	3	2	6
Gli-2	ES 23/02	19	Subventricular zone	2	2	4
Gli-2	ES 23/02	19	Ventricular zone	4	2	8
Gli-2	ES 22/02	19	Marginal zone	3	2	6
Gli-2	ES 22/02	19	Cortical plate	4	2	8
Gli-2	ES 22/02	19	Subplate	4	2	8
Gli-2	ES 22/02	19	Intermediate zone	3	3	9
Gli-2	ES 22/02	19	Subventricular zone	3	2	6
Gli-2	ES 22/02	19	Ventricular zone	3	2	6
Gli-2	ES 15/02	21	Marginal zone	2	2	4
Gli-2	ES 15/02	21	Cortical plate	4	2	8
Gli-2	ES 15/02	21	Subplate	3	2	6
Gli-2	ES 15/02	21	Intermediate zone	2	3	6
Gli-2	ES 15/02	21	Subventricular zone	3	2	6
Gli-2	ES 15/02	21	Ventricular zone	4	2	8
Gli-2	ES 4/02	19	Marginal zone			
Gli-2	ES 4/02	19	Cortical plate	4	2	8
Gli-2	ES 4/02	19	Subplate	4	2	8

Gli-2	ES 4/02	19	Intermediate zone	2	2	4
Gli-2	ES 4/02	19	Subventricular zone	2	3	6
Gli-2	ES 4/02	19	Ventricular zone	3	3	9
Gli-2	ES 154/01	18	Marginal zone	2	3	6
Gli-2	ES 154/01	18	Cortical plate	2	2	4
Gli-2	ES 154/01	18	Subplate	4	1	4
Gli-2	ES 154/01	18	Intermediate zone	2	2	4
Gli-2	ES 154/01	18	Subventricular zone	3	2	6
Gli-2	ES 154/01	18	Ventricular zone	4	2	8
Gli-2	ES 151/01	13	Marginal zone	3	3	9
Gli-2	ES 151/01	13	Cortical plate	3	3	9
Gli-2	ES 151/01	13	Subplate	3	2	6
Gli-2	ES 151/01	13	Intermediate zone	2	3	6
Gli-2	ES 151/01	13	Subventricular zone	3	2	6
Gli-2	ES 151/01	13	Ventricular zone	4	3	12
Gli-2	ES 129/01	26	Marginal zone	2	3	6
Gli-2	ES 129/01	26	Cortical plate	4	3	12
Gli-2	ES 129/01	26	Subplate	4	3	12
Gli-2	ES 129/01	26	Intermediate zone	2	3	6
Gli-2	ES 129/01	26	Subventricular zone	2	2	4
Gli-2	ES 129/01	26	Ventricular zone	2	2	4
Gli-3	ES 49/04	20	Marginal zone	2	3	6
Gli-3	ES 49/04	20	Cortical plate	2	2	4
Gli-3	ES 49/04	20	Subplate	2	2	4
Gli-3	ES 49/04	20	Intermediate zone	2	2	4
Gli-3	ES 49/04	20	Subventricular zone	3	3	9
Gli-3	ES 49/04	20	Ventricular zone			
Gli-3	ES 15/04	16	Marginal zone	2	3	6
Gli-3	ES 15/04	16	Cortical plate	3	3	9

Gli-3	ES 15/04	16	Subplate	3	3	9
Gli-3	ES 15/04	16	Intermediate zone	3	3	9
Gli-3	ES 15/04	16	Subventricular zone	2	3	6
Gli-3	ES 15/04	16	Ventricular zone	4	3	12
Gli-3	ES 3/04	14	Marginal zone	3	2	6
Gli-3	ES 3/04	14	Cortical plate	2	2	4
Gli-3	ES 3/04	14	Subplate	2	2	4
Gli-3	ES 3/04	14	Intermediate zone	2	1	2
Gli-3	ES 3/04	14	Subventricular zone	2	1	2
Gli-3	ES 3/04	14	Ventricular zone	3	2	6
Gli-3	ES 155/03	12	Marginal zone	1	2	2
Gli-3	ES 155/03	12	Cortical plate	2	2	4
Gli-3	ES 155/03	12	Subplate	1	2	2
Gli-3	ES 155/03	12	Intermediate zone	1	2	2
Gli-3	ES 155/03	12	Subventricular zone	2	2	4
Gli-3	ES 155/03	12	Ventricular zone	2	2	4
Gli-3	ES 148/03	22	Marginal zone	2	3	6
Gli-3	ES 148/03	22	Cortical plate	3	3	9
Gli-3	ES 148/03	22	Subplate	2	2	4
Gli-3	ES 148/03	22	Intermediate zone	1	2	2
Gli-3	ES 148/03	22	Subventricular zone	3	2	6
Gli-3	ES 148/03	22	Ventricular zone	4	3	12
Gli-3	ES 144/03	13	Marginal zone	4	3	12
Gli-3	ES 144/03	13	Cortical plate	3	3	9
Gli-3	ES 144/03	13	Subplate	3	3	9
Gli-3	ES 144/03	13	Intermediate zone	2	2	4
Gli-3	ES 144/03	13	Subventricular zone	2	2	4
Gli-3	ES 144/03	13	Ventricular zone	3	2	6
Gli-3	ES 142/03	14	Marginal zone		0	

Gli-3	ES 142/03	14	Cortical plate	3	3	9
Gli-3	ES 142/03	14	Subplate	2	2	4
Gli-3	ES 142/03	14	Intermediate zone	2	2	4
Gli-3	ES 142/03	14	Subventricular zone	3	2	6
Gli-3	ES 142/03	14	Ventricular zone	2	2	4
Gli-3	ES 128/03	18	Marginal zone	3	3	9
Gli-3	ES 128/03	18	Cortical plate	4	3	12
Gli-3	ES 128/03	18	Subplate	3	2	6
Gli-3	ES 128/03	18	Intermediate zone	3	2	6
Gli-3	ES 128/03	18	Subventricular zone	3	3	9
Gli-3	ES 128/03	18	Ventricular zone	4	3	12
Gli-3	ES 118/03	22	Marginal zone	2	3	6
Gli-3	ES 118/03	22	Cortical plate	3	3	9
Gli-3	ES 118/03	22	Subplate	2	2	4
Gli-3	ES 118/03	22	Intermediate zone	2	3	6
Gli-3	ES 118/03	22	Subventricular zone	2	2	4
Gli-3	ES 118/03	22	Ventricular zone	1	2	2
Gli-3	ES 112/03	21	Marginal zone	2	3	6
Gli-3	ES 112/03	21	Cortical plate	2	2	4
Gli-3	ES 112/03	21	Subplate	2	2	4
Gli-3	ES 112/03	21	Intermediate zone	2	2	4
Gli-3	ES 112/03	21	Subventricular zone	2	2	4
Gli-3	ES 112/03	21	Ventricular zone	3	2	6
Gli-3	ES 67/03	28	Marginal zone			
Gli-3	ES 67/03	28	Cortical plate			
Gli-3	ES 67/03	28	Subplate			
Gli-3	ES 67/03	28	Intermediate zone			
Gli-3	ES 67/03	28	Subventricular zone			
Gli-3	ES 67/03	28	Ventricular zone			

Gli-3	ES 67/03	28	Marginal zone	3	3	9
Gli-3	ES 67/03	28	Cortical plate	4	3	12
Gli-3	ES 67/03	28	Subplate	4	3	12
Gli-3	ES 67/03	28	Intermediate zone	2	3	6
Gli-3	ES 67/03	28	Subventricular zone	3	2	6
Gli-3	ES 67/03	28	Ventricular zone	3	2	6
Gli-3	ES 58/03	16	Marginal zone			
Gli-3	ES 58/03	16	Cortical plate			
Gli-3	ES 58/03	16	Subplate			
Gli-3	ES 58/03	16	Intermediate zone			
Gli-3	ES 58/03	16	Subventricular zone			
Gli-3	ES 58/03	16	Ventricular zone			
Gli-3	ES 187/02	14	Marginal zone	2	3	6
Gli-3	ES 187/02	14	Cortical plate	2	2	4
Gli-3	ES 187/02	14	Subplate	2	2	4
Gli-3	ES 187/02	14	Intermediate zone	2	2	4
Gli-3	ES 187/02	14	Subventricular zone	2	2	4
Gli-3	ES 187/02	14	Ventricular zone	2	2	4
Gli-3	ES 102/02	13	Marginal zone	3	3	9
Gli-3	ES 102/02	13	Cortical plate	3	2	6
Gli-3	ES 102/02	13	Subplate	2	2	4
Gli-3	ES 102/02	13	Intermediate zone	2	2	4
Gli-3	ES 102/02	13	Subventricular zone	2	2	4
Gli-3	ES 102/02	13	Ventricular zone	2	2	4
Gli-3	ES 38/02	17	Marginal zone			
Gli-3	ES 38/02	17	Cortical plate	4	3	12
Gli-3	ES 38/02	17	Subplate	3	3	9
Gli-3	ES 38/02	17	Intermediate zone	3	3	9
Gli-3	ES 38/02	17	Subventricular zone	3	2	6

Gli-3	ES 38/02	17	Ventricular zone	4	2	8
Gli-3	ES 23/02	19	Marginal zone	1	3	3
Gli-3	ES 23/02	19	Cortical plate	2	3	6
Gli-3	ES 23/02	19	Subplate	2	2	4
Gli-3	ES 23/02	19	Intermediate zone	2	2	4
Gli-3	ES 23/02	19	Subventricular zone	2	3	6
Gli-3	ES 23/02	19	Ventricular zone	3	3	9
Gli-3	ES 22/02	19	Marginal zone	3	3	9
Gli-3	ES 22/02	19	Cortical plate	2	2	4
Gli-3	ES 22/02	19	Subplate	3	2	6
Gli-3	ES 22/02	19	Intermediate zone	2	2	4
Gli-3	ES 22/02	19	Subventricular zone	3	3	9
Gli-3	ES 22/02	19	Ventricular zone	4	3	12
Gli-3	ES 15/02	21	Marginal zone	1	2	2
Gli-3	ES 15/02	21	Cortical plate	2	2	4
Gli-3	ES 15/02	21	Subplate	2	2	4
Gli-3	ES 15/02	21	Intermediate zone	2	2	4
Gli-3	ES 15/02	21	Subventricular zone	2	2	4
Gli-3	ES 15/02	21	Ventricular zone	3	2	6
Gli-3	ES 4/02	19	Marginal zone	2	2	4
Gli-3	ES 4/02	19	Cortical plate	3	2	6
Gli-3	ES 4/02	19	Subplate	2	3	6
Gli-3	ES 4/02	19	Intermediate zone	2	2	4
Gli-3	ES 4/02	19	Subventricular zone	3	3	9
Gli-3	ES 4/02	19	Ventricular zone	4	2	8
Gli-3	ES 154/01	18	Marginal zone	2	3	6
Gli-3	ES 154/01	18	Cortical plate	2	2	4
Gli-3	ES 154/01	18	Subplate	2	2	4
Gli-3	ES 154/01	18	Intermediate zone	2	1	2

Gli-3	ES 154/01	18	Subventricular zone	3	2	6
Gli-3	ES 154/01	18	Ventricular zone	4	2	8
Gli-3	ES 151/01	13	Marginal zone	2	2	4
Gli-3	ES 151/01	13	Cortical plate	4	2	8
Gli-3	ES 151/01	13	Subplate	3	1	3
Gli-3	ES 151/01	13	Intermediate zone	2	2	4
Gli-3	ES 151/01	13	Subventricular zone	2	2	4
Gli-3	ES 151/01	13	Ventricular zone	3	2	6
Gli-3	ES 129/01	26	Marginal zone	2	3	6
Gli-3	ES 129/01	26	Cortical plate	2	2	4
Gli-3	ES 129/01	26	Subplate	2	1	2
Gli-3	ES 129/01	26	Intermediate zone	2	2	4
Gli-3	ES 129/01	26	Subventricular zone			
Gli-3	ES 129/01	26	Ventricular zone			
SHH	ES 49/04	20	Marginal zone	2	2	4
SHH	ES 49/04	20	Cortical plate	3	3	9
SHH	ES 49/04	20	Subplate	2	3	6
SHH	ES 49/04	20	Intermediate zone	2	2	4
SHH	ES 49/04	20	Subventricular zone	2	2	4
SHH	ES 49/04	20	Ventricular zone	3	2	6
SHH	ES 15/04	16	Marginal zone	2	2	4
SHH	ES 15/04	16	Cortical plate	3	2	6
SHH	ES 15/04	16	Subplate	2	2	4
SHH	ES 15/04	16	Intermediate zone	2	2	4
SHH	ES 15/04	16	Subventricular zone	2	2	4
SHH	ES 15/04	16	Ventricular zone	4	2	8
SHH	ES 3/04	14	Marginal zone	2	2	4
SHH	ES 3/04	14	Cortical plate	3	3	9
SHH	ES 3/04	14	Subplate	3	2	6

SHH	ES 3/04	14	Intermediate zone	3	2	6
SHH	ES 3/04	14	Subventricular zone	2	3	6
SHH	ES 3/04	14	Ventricular zone	4	3	12
SHH	ES 155/03	12	Marginal zone	2	2	4
SHH	ES 155/03	12	Cortical plate	4	2	8
SHH	ES 155/03	12	Subplate	4	2	8
SHH	ES 155/03	12	Intermediate zone	2	2	4
SHH	ES 155/03	12	Subventricular zone	3	2	6
SHH	ES 155/03	12	Ventricular zone	3	3	9
SHH	ES 148/03	22	Marginal zone	2	2	4
SHH	ES 148/03	22	Cortical plate	3	3	9
SHH	ES 148/03	22	Subplate	2	3	6
SHH	ES 148/03	22	Intermediate zone	2	1	2
SHH	ES 148/03	22	Subventricular zone	2	3	6
SHH	ES 148/03	22	Ventricular zone	2	3	6
SHH	ES 144/03	13	Marginal zone	2	3	6
SHH	ES 144/03	13	Cortical plate	4	3	12
SHH	ES 144/03	13	Subplate	2	3	6
SHH	ES 144/03	13	Intermediate zone	2	2	4
SHH	ES 144/03	13	Subventricular zone	2	3	6
SHH	ES 144/03	13	Ventricular zone	3	3	9
SHH	ES 142/03	14	Marginal zone			
SHH	ES 142/03	14	Cortical plate	4	2	8
SHH	ES 142/03	14	Subplate	2	2	4
SHH	ES 142/03	14	Intermediate zone	2	1	2
SHH	ES 142/03	14	Subventricular zone	2	3	6
SHH	ES 142/03	14	Ventricular zone	2	3	6
SHH	ES 128/03	18	Marginal zone		0	
SHH	ES 128/03	18	Cortical plate	2	1	2

SHH	ES 128/03	18	Subplate		0	
SHH	ES 128/03	18	Intermediate zone		0	
SHH	ES 128/03	18	Subventricular zone	2	1	2
SHH	ES 128/03	18	Ventricular zone	4	1	4
SHH	ES 118/03	22	Marginal zone	3	2	6
SHH	ES 118/03	22	Cortical plate	3	2	6
SHH	ES 118/03	22	Subplate	2	2	4
SHH	ES 118/03	22	Intermediate zone	2	3	6
SHH	ES 118/03	22	Subventricular zone	1	2	2
SHH	ES 118/03	22	Ventricular zone	2	2	4
SHH	ES 112/03	21	Marginal zone	2	2	4
SHH	ES 112/03	21	Cortical plate	2	3	6
SHH	ES 112/03	21	Subplate	3	3	9
SHH	ES 112/03	21	Intermediate zone	2	2	4
SHH	ES 112/03	21	Subventricular zone	2	2	4
SHH	ES 112/03	21	Ventricular zone	3	2	6
SHH	ES 67/03	28	Marginal zone	2	2	4
SHH	ES 67/03	28	Cortical plate	2	3	6
SHH	ES 67/03	28	Subplate	2	3	6
SHH	ES 67/03	28	Intermediate zone	2	3	6
SHH	ES 67/03	28	Subventricular zone	2	2	4
SHH	ES 67/03	28	Ventricular zone	3	2	6
SHH	ES 67/03	28	Marginal zone	1	3	3
SHH	ES 67/03	28	Cortical plate	4	3	12
SHH	ES 67/03	28	Subplate	3	3	9
SHH	ES 67/03	28	Intermediate zone	1	3	3
SHH	ES 67/03	28	Subventricular zone	2	2	4
SHH	ES 67/03	28	Ventricular zone	2	2	4
SHH	ES 58/03	16	Marginal zone			

SHH	ES 58/03	16	Cortical plate			
SHH	ES 58/03	16	Subplate			
SHH	ES 58/03	16	Intermediate zone			
SHH	ES 58/03	16	Subventricular zone			
SHH	ES 58/03	16	Ventricular zone			
SHH	ES 187/02	14	Marginal zone	2	2	4
SHH	ES 187/02	14	Cortical plate	4	2	8
SHH	ES 187/02	14	Subplate	3	2	6
SHH	ES 187/02	14	Intermediate zone	2	2	4
SHH	ES 187/02	14	Subventricular zone	3	3	9
SHH	ES 187/02	14	Ventricular zone	3	3	9
SHH	ES 102/02	13	Marginal zone	1	2	2
SHH	ES 102/02	13	Cortical plate	3	2	6
SHH	ES 102/02	13	Subplate	2	2	4
SHH	ES 102/02	13	Intermediate zone	1	2	2
SHH	ES 102/02	13	Subventricular zone	1	3	3
SHH	ES 102/02	13	Ventricular zone	3	3	9
SHH	ES 38/02	17	Marginal zone			
SHH	ES 38/02	17	Cortical plate	3	2	6
SHH	ES 38/02	17	Subplate	3	2	6
SHH	ES 38/02	17	Intermediate zone	3	1	3
SHH	ES 38/02	17	Subventricular zone	4	1	4
SHH	ES 38/02	17	Ventricular zone	3	1	3
SHH	ES 23/02	19	Marginal zone	3	2	6
SHH	ES 23/02	19	Cortical plate	3	2	6
SHH	ES 23/02	19	Subplate	3	2	6
SHH	ES 23/02	19	Intermediate zone	3	2	6
SHH	ES 23/02	19	Subventricular zone	3	2	6
SHH	ES 23/02	19	Ventricular zone	4	2	8

SHH	ES 22/02	19	Marginal zone	2	2	4
SHH	ES 22/02	19	Cortical plate	4	2	8
SHH	ES 22/02	19	Subplate	3	2	6
SHH	ES 22/02	19	Intermediate zone	2	2	4
SHH	ES 22/02	19	Subventricular zone	3	2	6
SHH	ES 22/02	19	Ventricular zone	3	3	9
SHH	ES 15/02	21	Marginal zone	2	2	4
SHH	ES 15/02	21	Cortical plate	3	2	6
SHH	ES 15/02	21	Subplate	3	2	6
SHH	ES 15/02	21	Intermediate zone	2	2	4
SHH	ES 15/02	21	Subventricular zone	2	2	4
SHH	ES 15/02	21	Ventricular zone	2	3	6
SHH	ES 4/02	19	Marginal zone			
SHH	ES 4/02	19	Cortical plate	4	2	8
SHH	ES 4/02	19	Subplate	3	2	6
SHH	ES 4/02	19	Intermediate zone	2	2	4
SHH	ES 4/02	19	Subventricular zone	3	2	6
SHH	ES 4/02	19	Ventricular zone	4	2	8
SHH	ES 154/01	18	Marginal zone	2	3	6
SHH	ES 154/01	18	Cortical plate	3	3	9
SHH	ES 154/01	18	Subplate	3	3	9
SHH	ES 154/01	18	Intermediate zone	3	2	6
SHH	ES 154/01	18	Subventricular zone	2	3	6
SHH	ES 154/01	18	Ventricular zone	4	3	12
SHH	ES 151/01	13	Marginal zone			
SHH	ES 151/01	13	Cortical plate	4	2	8
SHH	ES 151/01	13	Subplate	4	2	8
SHH	ES 151/01	13	Intermediate zone	1	3	3
SHH	ES 151/01	13	Subventricular zone	4	2	8

SHH	ES 151/01	13	Ventricular zone	3	3	9
SHH	ES 129/01	26	Marginal zone	2	2	4
SHH	ES 129/01	26	Cortical plate	3	2	6
SHH	ES 129/01	26	Subplate	3	2	6
SHH	ES 129/01	26	Intermediate zone	2	1	2
SHH	ES 129/01	26	Subventricular zone	3	2	6
SHH	ES 129/01	26	Ventricular zone	3	2	6
patched	ES 49/04	20	Marginal zone	2	2	4
patched	ES 49/04	20	Cortical plate	3	2	6
patched	ES 49/04	20	Subplate	3	2	6
patched	ES 49/04	20	Intermediate zone	3	2	6
patched	ES 49/04	20	Subventricular zone	3	2	6
patched	ES 49/04	20	Ventricular zone	4	2	8
patched	ES 15/04	16	Marginal zone	2	2	4
patched	ES 15/04	16	Cortical plate	4	2	8
patched	ES 15/04	16	Subplate	3	2	6
patched	ES 15/04	16	Intermediate zone	2	2	4
patched	ES 15/04	16	Subventricular zone	3	2	6
patched	ES 15/04	16	Ventricular zone	4	2	8
patched	ES 3/04	14	Marginal zone	2	2	4
patched	ES 3/04	14	Cortical plate	2	3	6
patched	ES 3/04	14	Subplate	1	3	3
patched	ES 3/04	14	Intermediate zone	1	2	2
patched	ES 3/04	14	Subventricular zone	2	2	4
patched	ES 3/04	14	Ventricular zone	3	2	6
patched	ES 155/03	12	Marginal zone	2	3	6
patched	ES 155/03	12	Cortical plate	3	2	6
patched	ES 155/03	12	Subplate	2	2	4
patched	ES 155/03	12	Intermediate zone	2	2	4

patched	ES 155/03	12	Subventricular zone	3	2	6
patched	ES 155/03	12	Ventricular zone	4	2	8
patched	ES 148/03	22	Marginal zone	1	2	2
patched	ES 148/03	22	Cortical plate	3	2	6
patched	ES 148/03	22	Subplate	2	2	4
patched	ES 148/03	22	Intermediate zone	3	2	6
patched	ES 148/03	22	Subventricular zone	2	2	4
patched	ES 148/03	22	Ventricular zone	2	2	4
patched	ES 144/03	13	Marginal zone	3	3	9
patched	ES 144/03	13	Cortical plate	2	2	4
patched	ES 144/03	13	Subplate	2	2	4
patched	ES 144/03	13	Intermediate zone	3	1	3
patched	ES 144/03	13	Subventricular zone	4	2	8
patched	ES 144/03	13	Ventricular zone	4	2	8
patched	ES 142/03	14	Marginal zone			
patched	ES 142/03	14	Cortical plate	4	2	8
patched	ES 142/03	14	Subplate	3	2	6
patched	ES 142/03	14	Intermediate zone	2	2	4
patched	ES 142/03	14	Subventricular zone	3	2	6
patched	ES 142/03	14	Ventricular zone	4	2	8
patched	ES 128/03	18	Marginal zone	2	2	4
patched	ES 128/03	18	Cortical plate	2	2	4
patched	ES 128/03	18	Subplate	2	2	4
patched	ES 128/03	18	Intermediate zone	2	2	4
patched	ES 128/03	18	Subventricular zone	3	2	6
patched	ES 128/03	18	Ventricular zone	4	2	8
patched	ES 118/03	22	Marginal zone	2	2	4
patched	ES 118/03	22	Cortical plate	4	2	8
patched	ES 118/03	22	Subplate	3	2	6

patched	ES 118/03	22	Intermediate zone	3	3	9
patched	ES 118/03	22	Subventricular zone	2	2	4
patched	ES 118/03	22	Ventricular zone	2	2	4
patched	ES 112/03	21	Marginal zone	2	2	4
patched	ES 112/03	21	Cortical plate	4	2	8
patched	ES 112/03	21	Subplate	3	3	9
patched	ES 112/03	21	Intermediate zone	3	2	6
patched	ES 112/03	21	Subventricular zone	3	2	6
patched	ES 112/03	21	Ventricular zone	4	2	8
patched	ES 67/03	28	Marginal zone	2	3	6
patched	ES 67/03	28	Cortical plate	2	2	4
patched	ES 67/03	28	Subplate	3	2	6
patched	ES 67/03	28	Intermediate zone	3	2	6
patched	ES 67/03	28	Subventricular zone	2	2	4
patched	ES 67/03	28	Ventricular zone	3	2	6
patched	ES 67/03	28	Marginal zone	4	3	12
patched	ES 67/03	28	Cortical plate	2	2	4
patched	ES 67/03	28	Subplate	3	2	6
patched	ES 67/03	28	Intermediate zone	3	2	6
patched	ES 67/03	28	Subventricular zone	2	2	4
patched	ES 67/03	28	Ventricular zone	2	2	4
patched	ES 58/03	16	Marginal zone			
patched	ES 58/03	16	Cortical plate			
patched	ES 58/03	16	Subplate			
patched	ES 58/03	16	Intermediate zone			
patched	ES 58/03	16	Subventricular zone			
patched	ES 58/03	16	Ventricular zone			
patched	ES 187/02	14	Marginal zone	2	2	4
patched	ES 187/02	14	Cortical plate	4	1	4

patched	ES 187/02	14	Subplate	4	1	4
patched	ES 187/02	14	Intermediate zone	2	2	4
patched	ES 187/02	14	Subventricular zone	2	2	4
patched	ES 187/02	14	Ventricular zone	4	2	8
patched	ES 102/02	13	Marginal zone	4	3	12
patched	ES 102/02	13	Cortical plate	3	3	9
patched	ES 102/02	13	Subplate	3	3	9
patched	ES 102/02	13	Intermediate zone	2	2	4
patched	ES 102/02	13	Subventricular zone	3	2	6
patched	ES 102/02	13	Ventricular zone	4	2	8
patched	ES 38/02	17	Marginal zone	1	3	3
patched	ES 38/02	17	Cortical plate	3	2	6
patched	ES 38/02	17	Subplate	2	2	4
patched	ES 38/02	17	Intermediate zone	2	2	4
patched	ES 38/02	17	Subventricular zone	3	2	6
patched	ES 38/02	17	Ventricular zone	4	2	8
patched	ES 23/02	19	Marginal zone	3	3	9
patched	ES 23/02	19	Cortical plate	2	3	6
patched	ES 23/02	19	Subplate	3	3	9
patched	ES 23/02	19	Intermediate zone	2	2	4
patched	ES 23/02	19	Subventricular zone	2	2	4
patched	ES 23/02	19	Ventricular zone	4	2	8
patched	ES 22/02	19	Marginal zone	3	3	9
patched	ES 22/02	19	Cortical plate	4	3	12
patched	ES 22/02	19	Subplate	4	2	8
patched	ES 22/02	19	Intermediate zone	4	2	8
patched	ES 22/02	19	Subventricular zone	3	2	6
patched	ES 22/02	19	Ventricular zone	3	2	6
patched	ES 15/02	21	Marginal zone	2	2	4

patched	ES 15/02	21	Cortical plate	2	3	6
patched	ES 15/02	21	Subplate	3	3	9
patched	ES 15/02	21	Intermediate zone	2	3	6
patched	ES 15/02	21	Subventricular zone	2	2	4
patched	ES 15/02	21	Ventricular zone	2	2	4
patched	ES 4/02	19	Marginal zone	4	3	12
patched	ES 4/02	19	Cortical plate	4	2	8
patched	ES 4/02	19	Subplate	2	3	6
patched	ES 4/02	19	Intermediate zone	2	2	4
patched	ES 4/02	19	Subventricular zone	2	2	4
patched	ES 4/02	19	Ventricular zone	3	2	6
patched	ES 154/01	18	Marginal zone	2	3	6
patched	ES 154/01	18	Cortical plate	4	2	8
patched	ES 154/01	18	Subplate	4	2	8
patched	ES 154/01	18	Intermediate zone	3	2	6
patched	ES 154/01	18	Subventricular zone	2	3	6
patched	ES 154/01	18	Ventricular zone	4	3	12
patched	ES 151/01	13	Marginal zone	2	4	8
patched	ES 151/01	13	Cortical plate	2	3	6
patched	ES 151/01	13	Subplate	4	2	8
patched	ES 151/01	13	Intermediate zone	2	2	4
patched	ES 151/01	13	Subventricular zone	3	2	6
patched	ES 151/01	13	Ventricular zone	4	2	8
patched	ES 129/01	26	Marginal zone	2	3	6
patched	ES 129/01	26	Cortical plate	4	3	12
patched	ES 129/01	26	Subplate	2	3	6
patched	ES 129/01	26	Intermediate zone	2	3	6
patched	ES 129/01	26	Subventricular zone	1	3	3
patched	ES 129/01	26	Ventricular zone	2	3	6

Smoothened	ES 49/04	20	Marginal zone	2	2	4
Smoothened	ES 49/04	20	Cortical plate	2	2	4
Smoothened	ES 49/04	20	Subplate	3	2	6
Smoothened	ES 49/04	20	Intermediate zone	2	2	4
Smoothened	ES 49/04	20	Subventricular zone	4	2	8
Smoothened	ES 49/04	20	Ventricular zone	4	1	4
Smoothened	ES 15/04	16	Marginal zone	2	1	2
Smoothened	ES 15/04	16	Cortical plate	2	1	2
Smoothened	ES 15/04	16	Subplate	1	1	1
Smoothened	ES 15/04	16	Intermediate zone	1	1	1
Smoothened	ES 15/04	16	Subventricular zone	2	1	2
Smoothened	ES 15/04	16	Ventricular zone	2	1	2
Smoothened	ES 3/04	14	Marginal zone	2	2	4
Smoothened	ES 3/04	14	Cortical plate	3	2	6
Smoothened	ES 3/04	14	Subplate	3	2	6
Smoothened	ES 3/04	14	Intermediate zone	3	2	6
Smoothened	ES 3/04	14	Subventricular zone	2	2	4
Smoothened	ES 3/04	14	Ventricular zone	4	2	8
Smoothened	ES 155/03	12	Marginal zone	2	1	2
Smoothened	ES 155/03	12	Cortical plate	3	2	6
Smoothened	ES 155/03	12	Subplate	3	2	6
Smoothened	ES 155/03	12	Intermediate zone	3	2	6
Smoothened	ES 155/03	12	Subventricular zone	4	1	4
Smoothened	ES 155/03	12	Ventricular zone	4	3	12
Smoothened	ES 148/03	22	Marginal zone	1	1	1
Smoothened	ES 148/03	22	Cortical plate	3	2	6
Smoothened	ES 148/03	22	Subplate	3	2	6
Smoothened	ES 148/03	22	Intermediate zone	2	1	2
Smoothened	ES 148/03	22	Subventricular zone	2	2	4

Smoothened	ES 148/03	22	Ventricular zone	3	2	6
Smoothened	ES 144/03	13	Marginal zone	2	2	4
Smoothened	ES 144/03	13	Cortical plate	2	2	4
Smoothened	ES 144/03	13	Subplate	3	2	6
Smoothened	ES 144/03	13	Intermediate zone	2	1	2
Smoothened	ES 144/03	13	Subventricular zone	3	1	3
Smoothened	ES 144/03	13	Ventricular zone	4	2	8
Smoothened	ES 142/03	14	Marginal zone			
Smoothened	ES 142/03	14	Cortical plate	3	2	6
Smoothened	ES 142/03	14	Subplate	2	2	4
Smoothened	ES 142/03	14	Intermediate zone	2	1	2
Smoothened	ES 142/03	14	Subventricular zone	2	2	4
Smoothened	ES 142/03	14	Ventricular zone	3	2	6
Smoothened	ES 128/03	18	Marginal zone	3	2	6
Smoothened	ES 128/03	18	Cortical plate	2	2	4
Smoothened	ES 128/03	18	Subplate	2	2	4
Smoothened	ES 128/03	18	Intermediate zone	2	1	2
Smoothened	ES 128/03	18	Subventricular zone	3	2	6
Smoothened	ES 128/03	18	Ventricular zone	4	2	8
Smoothened	ES 118/03	22	Marginal zone	2	2	4
Smoothened	ES 118/03	22	Cortical plate	3	2	6
Smoothened	ES 118/03	22	Subplate			
Smoothened	ES 118/03	22	Intermediate zone			
Smoothened	ES 118/03	22	Subventricular zone			
Smoothened	ES 118/03	22	Ventricular zone			
Smoothened	ES 112/03	21	Marginal zone	3	2	6
Smoothened	ES 112/03	21	Cortical plate	2	2	4
Smoothened	ES 112/03	21	Subplate	3	2	6
Smoothened	ES 112/03	21	Intermediate zone	2	2	4

Smoothened	ES 112/03	21	Subventricular zone	2	2	4
Smoothened	ES 112/03	21	Ventricular zone	4	2	8
Smoothened	ES 67/03	28	Marginal zone	2	2	4
Smoothened	ES 67/03	28	Cortical plate	3	2	6
Smoothened	ES 67/03	28	Subplate	2	2	4
Smoothened	ES 67/03	28	Intermediate zone	3	2	6
Smoothened	ES 67/03	28	Subventricular zone			
Smoothened	ES 67/03	28	Ventricular zone			
Smoothened	ES 67/03	28	Marginal zone	2	3	6
Smoothened	ES 67/03	28	Cortical plate	4	3	12
Smoothened	ES 67/03	28	Subplate	3	3	9
Smoothened	ES 67/03	28	Intermediate zone	3	2	6
Smoothened	ES 67/03	28	Subventricular zone	2	2	4
Smoothened	ES 67/03	28	Ventricular zone			
Smoothened	ES 58/03	16	Marginal zone	2	2	4
Smoothened	ES 58/03	16	Cortical plate	4	2	8
Smoothened	ES 58/03	16	Subplate	3	2	6
Smoothened	ES 58/03	16	Intermediate zone	3	2	6
Smoothened	ES 58/03	16	Subventricular zone	3	2	6
Smoothened	ES 58/03	16	Ventricular zone	2	2	4
Smoothened	ES 187/02	14	Marginal zone	2	2	4
Smoothened	ES 187/02	14	Cortical plate	3	2	6
Smoothened	ES 187/02	14	Subplate	3	2	6
Smoothened	ES 187/02	14	Intermediate zone	2	2	4
Smoothened	ES 187/02	14	Subventricular zone	2	2	4
Smoothened	ES 187/02	14	Ventricular zone	4	2	8
Smoothened	ES 102/02	13	Marginal zone	2	2	4
Smoothened	ES 102/02	13	Cortical plate	4	2	8
Smoothened	ES 102/02	13	Subplate	3	2	6

Smoothened	ES 102/02	13	Intermediate zone	2	2	4
Smoothened	ES 102/02	13	Subventricular zone	3	2	6
Smoothened	ES 102/02	13	Ventricular zone	4	2	8
Smoothened	ES 38/02	17	Marginal zone			
Smoothened	ES 38/02	17	Cortical plate	2	2	4
Smoothened	ES 38/02	17	Subplate	2	2	4
Smoothened	ES 38/02	17	Intermediate zone	2	2	4
Smoothened	ES 38/02	17	Subventricular zone	3	2	6
Smoothened	ES 38/02	17	Ventricular zone	4	2	8
Smoothened	ES 23/02	19	Marginal zone	2	2	4
Smoothened	ES 23/02	19	Cortical plate	2	2	4
Smoothened	ES 23/02	19	Subplate	2	2	4
Smoothened	ES 23/02	19	Intermediate zone	2	1	2
Smoothened	ES 23/02	19	Subventricular zone	2	2	4
Smoothened	ES 23/02	19	Ventricular zone	4	1	4
Smoothened	ES 22/02	19	Marginal zone	2	2	4
Smoothened	ES 22/02	19	Cortical plate	4	2	8
Smoothened	ES 22/02	19	Subplate	3	2	6
Smoothened	ES 22/02	19	Intermediate zone	2	2	4
Smoothened	ES 22/02	19	Subventricular zone	3	2	6
Smoothened	ES 22/02	19	Ventricular zone	3	2	6
Smoothened	ES 15/02	21	Marginal zone	1	2	2
Smoothened	ES 15/02	21	Cortical plate	3	2	6
Smoothened	ES 15/02	21	Subplate	3	2	6
Smoothened	ES 15/02	21	Intermediate zone	2	2	4
Smoothened	ES 15/02	21	Subventricular zone	3	2	6
Smoothened	ES 15/02	21	Ventricular zone	4	2	8
Smoothened	ES 4/02	19	Marginal zone	2	2	4
Smoothened	ES 4/02	19	Cortical plate	4	2	8

Smoothened	ES 4/02	19	Subplate	3	2	6
Smoothened	ES 4/02	19	Intermediate zone	2	2	4
Smoothened	ES 4/02	19	Subventricular zone	2	2	4
Smoothened	ES 4/02	19	Ventricular zone	3	2	6
Smoothened	ES 154/01	18	Marginal zone	1	2	2
Smoothened	ES 154/01	18	Cortical plate	4	2	8
Smoothened	ES 154/01	18	Subplate	4	1	4
Smoothened	ES 154/01	18	Intermediate zone	2	1	2
Smoothened	ES 154/01	18	Subventricular zone	2	1	2
Smoothened	ES 154/01	18	Ventricular zone	2	2	4
Smoothened	ES 151/01	13	Marginal zone	2	2	4
Smoothened	ES 151/01	13	Cortical plate	4	2	8
Smoothened	ES 151/01	13	Subplate	4	2	8
Smoothened	ES 151/01	13	Intermediate zone	2	2	4
Smoothened	ES 151/01	13	Subventricular zone	2	2	4
Smoothened	ES 151/01	13	Ventricular zone	4	2	8
Smoothened	ES 129/01	26	Marginal zone	2	2	4
Smoothened	ES 129/01	26	Cortical plate	3	2	6
Smoothened	ES 129/01	26	Subplate	3	2	6
Smoothened	ES 129/01	26	Intermediate zone	2	2	4
Smoothened	ES 129/01	26	Subventricular zone	3	2	6
Smoothened	ES 129/01	26	Ventricular zone	2	2	4

Supplementary Table 2: Protein expression raw data for staining frequency, staining intensity and the resulting multi-score in the cerebellar layers (empty boxes: data acquisition was not possible due to missing tissue specimens or other technical reasons).

Factor	Case-ID	Week of gestation	Area	Frequency	Intensity	Multi-Score
Gli-1	ES 49/04	20	External granular layer	2	1	2
Gli-1	ES 49/04	20	Molecular layer	2	2	4
Gli-1	ES 49/04	20	Internal granular layer	3	2	6
Gli-1	ES 15/04	16	External granular layer			
Gli-1	ES 15/04	16	Molecular layer			
Gli-1	ES 15/04	16	Internal granular layer			
Gli-1	ES 3/04	14	External granular layer	3	1	3
Gli-1	ES 3/04	14	Molecular layer	1	2	2
Gli-1	ES 3/04	14	Internal granular layer	3	2	6
Gli-1	ES 155/03	12	External granular layer	4	3	12
Gli-1	ES 155/03	12	Molecular layer	2	2	4
Gli-1	ES 155/03	12	Internal granular layer	3	2	6
Gli-1	ES 148/03	22	External granular layer	1	1	1
Gli-1	ES 148/03	22	Molecular layer	1	1	1
Gli-1	ES 148/03	22	Internal granular layer	4	2	8
Gli-1	ES 144/03	13	External granular layer	4	3	12
Gli-1	ES 144/03	13	Molecular layer	2	2	4
Gli-1	ES 144/03	13	Internal granular layer	4	2	8
Gli-1	ES 142/03	14	External granular layer	4	3	12
Gli-1	ES 142/03	14	Molecular layer	2	2	4
Gli-1	ES 142/03	14	Internal granular layer	3	2	6
Gli-1	ES 128/03	18	External granular layer	3	3	9
Gli-1	ES 128/03	18	Molecular layer	2	2	4
Gli-1	ES 128/03	18	Internal granular layer	4	2	8

Gli-1	ES 118/03	22	External granular layer	3	2	6
Gli-1	ES 118/03	22	Molecular layer	1	1	1
Gli-1	ES 118/03	22	Internal granular layer	3	2	6
Gli-1	ES 112/03	21	External granular layer	2	2	4
Gli-1	ES 112/03	21	Molecular layer	1	2	2
Gli-1	ES 112/03	21	Internal granular layer	4	3	12
Gli-1	ES 67/03	28	External granular layer	1	1	1
Gli-1	ES 67/03	28	Molecular layer	1	1	1
Gli-1	ES 67/03	28	Internal granular layer	2	2	4
Gli-1	ES 67/03	28	External granular layer			
Gli-1	ES 67/03	28	Molecular layer			
Gli-1	ES 67/03	28	Internal granular layer			
Gli-1	ES 58/03	16	External granular layer	3	2	6
Gli-1	ES 58/03	16	Molecular layer	1	2	2
Gli-1	ES 58/03	16	Internal granular layer	3	2	6
Gli-1	ES 187/02	14	External granular layer			
Gli-1	ES 187/02	14	Molecular layer			
Gli-1	ES 187/02	14	Internal granular layer			
Gli-1	ES 102/02	13	External granular layer	4	3	12
Gli-1	ES 102/02	13	Molecular layer	3	2	6
Gli-1	ES 102/02	13	Internal granular layer	4	3	12
Gli-1	ES 38/02	17	External granular layer	1	2	2
Gli-1	ES 38/02	17	Molecular layer	2	1	2
Gli-1	ES 38/02	17	Internal granular layer	3	2	6
Gli-1	ES 23/02	19	External granular layer	4	3	12
Gli-1	ES 23/02	19	Molecular layer	2	2	4
Gli-1	ES 23/02	19	Internal granular layer	4	3	12
Gli-1	ES 22/02	19	External granular layer	3	1	3
Gli-1	ES 22/02	19	Molecular layer	1	1	1

Gli-1	ES 22/02	19	Internal granular layer	2	2	4
Gli-1	ES 15/02	21	External granular layer	3	2	6
Gli-1	ES 15/02	21	Molecular layer	2	1	2
Gli-1	ES 15/02	21	Internal granular layer	3	2	6
Gli-1	ES 4/02	19	External granular layer	3	2	6
Gli-1	ES 4/02	19	Molecular layer	1	1	1
Gli-1	ES 4/02	19	Internal granular layer	2	1	2
Gli-1	ES 154/01	18	External granular layer	2	2	4
Gli-1	ES 154/01	18	Molecular layer	1	1	1
Gli-1	ES 154/01	18	Internal granular layer	3	2	6
Gli-1	ES 151/01	13	External granular layer	3	2	6
Gli-1	ES 151/01	13	Molecular layer	1	2	2
Gli-1	ES 151/01	13	Internal granular layer	2	2	4
Gli-1	ES 129/01	26	External granular layer	2	2	4
Gli-1	ES 129/01	26	Molecular layer	1	1	1
Gli-1	ES 129/01	26	Internal granular layer	3	2	6
Gli-2	ES 49/04	20	External granular layer	3	3	9
Gli-2	ES 49/04	20	Molecular layer	2	3	6
Gli-2	ES 49/04	20	Internal granular layer	3	3	9
Gli-2	ES 15/04	16	External granular layer	3	3	9
Gli-2	ES 15/04	16	Molecular layer	2	3	6
Gli-2	ES 15/04	16	Internal granular layer	4	2	8
Gli-2	ES 3/04	14	External granular layer	4	3	12
Gli-2	ES 3/04	14	Molecular layer	2	2	4
Gli-2	ES 3/04	14	Internal granular layer	4	2	8
Gli-2	ES 155/03	12	External granular layer	4	3	12
Gli-2	ES 155/03	12	Molecular layer	2	2	4
Gli-2	ES 155/03	12	Internal granular layer	3	2	6
Gli-2	ES 148/03	22	External granular layer	2	3	6

Gli-2	ES 148/03	22	Molecular layer	3	2	6
Gli-2	ES 148/03	22	Internal granular layer	4	3	12
Gli-2	ES 144/03	13	External granular layer	4	3	12
Gli-2	ES 144/03	13	Molecular layer	3	3	9
Gli-2	ES 144/03	13	Internal granular layer	3	3	9
Gli-2	ES 142/03	14	External granular layer	4	3	12
Gli-2	ES 142/03	14	Molecular layer	3	2	6
Gli-2	ES 142/03	14	Internal granular layer	4	2	8
Gli-2	ES 128/03	18	External granular layer	4	3	12
Gli-2	ES 128/03	18	Molecular layer	2	3	6
Gli-2	ES 128/03	18	Internal granular layer	3	3	9
Gli-2	ES 118/03	22	External granular layer	2	2	4
Gli-2	ES 118/03	22	Molecular layer	2	2	4
Gli-2	ES 118/03	22	Internal granular layer	4	1	4
Gli-2	ES 112/03	21	External granular layer	3	3	9
Gli-2	ES 112/03	21	Molecular layer	2	2	4
Gli-2	ES 112/03	21	Internal granular layer	3	3	9
Gli-2	ES 67/03	28	External granular layer	4	3	12
Gli-2	ES 67/03	28	Molecular layer	2	3	6
Gli-2	ES 67/03	28	Internal granular layer	3	3	9
Gli-2	ES 67/03	28	External granular layer			
Gli-2	ES 67/03	28	Molecular layer			
Gli-2	ES 67/03	28	Internal granular layer			
Gli-2	ES 58/03	16	External granular layer	4	3	12
Gli-2	ES 58/03	16	Molecular layer	2	2	4
Gli-2	ES 58/03	16	Internal granular layer	3	2	6
Gli-2	ES 187/02	14	External granular layer			
Gli-2	ES 187/02	14	Molecular layer			
Gli-2	ES 187/02	14	Internal granular layer			

Gli-2	ES 102/02	13	External granular layer	4	2	8
Gli-2	ES 102/02	13	Molecular layer	3	2	6
Gli-2	ES 102/02	13	Internal granular layer	2	2	4
Gli-2	ES 38/02	17	External granular layer	4	3	12
Gli-2	ES 38/02	17	Molecular layer	2	3	6
Gli-2	ES 38/02	17	Internal granular layer	3	3	9
Gli-2	ES 23/02	19	External granular layer	4	3	12
Gli-2	ES 23/02	19	Molecular layer	4	2	8
Gli-2	ES 23/02	19	Internal granular layer	3	2	6
Gli-2	ES 22/02	19	External granular layer	4	3	12
Gli-2	ES 22/02	19	Molecular layer	2	3	6
Gli-2	ES 22/02	19	Internal granular layer	4	3	12
Gli-2	ES 15/02	21	External granular layer	3	3	9
Gli-2	ES 15/02	21	Molecular layer	2	2	4
Gli-2	ES 15/02	21	Internal granular layer	3	2	6
Gli-2	ES 4/02	19	External granular layer	4	3	12
Gli-2	ES 4/02	19	Molecular layer	4	1	4
Gli-2	ES 4/02	19	Internal granular layer	2	2	4
Gli-2	ES 154/01	18	External granular layer	4	3	12
Gli-2	ES 154/01	18	Molecular layer	3	2	6
Gli-2	ES 154/01	18	Internal granular layer	4	2	8
Gli-2	ES 151/01	13	External granular layer	4	3	12
Gli-2	ES 151/01	13	Molecular layer	2	3	6
Gli-2	ES 151/01	13	Internal granular layer	4	2	8
Gli-2	ES 129/01	26	External granular layer	4	3	12
Gli-2	ES 129/01	26	Molecular layer	2	3	6
Gli-2	ES 129/01	26	Internal granular layer	3	3	9
Gli-3	ES 49/04	20	External granular layer	3	3	9
Gli-3	ES 49/04	20	Molecular layer	2	3	6

Gli-3	ES 49/04	20	Internal granular layer	2	3	6
Gli-3	ES 15/04	16	External granular layer	4	3	12
Gli-3	ES 15/04	16	Molecular layer	3	3	9
Gli-3	ES 15/04	16	Internal granular layer	2	3	6
Gli-3	ES 3/04	14	External granular layer	4	3	12
Gli-3	ES 3/04	14	Molecular layer	2	2	4
Gli-3	ES 3/04	14	Internal granular layer	2	2	4
Gli-3	ES 155/03	12	External granular layer	4	3	12
Gli-3	ES 155/03	12	Molecular layer	2	2	4
Gli-3	ES 155/03	12	Internal granular layer	2	2	4
Gli-3	ES 148/03	22	External granular layer	4	3	12
Gli-3	ES 148/03	22	Molecular layer	2	3	6
Gli-3	ES 148/03	22	Internal granular layer	3	3	9
Gli-3	ES 144/03	13	External granular layer	3	2	6
Gli-3	ES 144/03	13	Molecular layer	2	3	6
Gli-3	ES 144/03	13	Internal granular layer	3	3	9
Gli-3	ES 142/03	14	External granular layer	4	3	12
Gli-3	ES 142/03	14	Molecular layer	3	2	6
Gli-3	ES 142/03	14	Internal granular layer	2	2	4
Gli-3	ES 128/03	18	External granular layer	4	3	12
Gli-3	ES 128/03	18	Molecular layer	4	3	12
Gli-3	ES 128/03	18	Internal granular layer	4	3	12
Gli-3	ES 118/03	22	External granular layer	3	3	9
Gli-3	ES 118/03	22	Molecular layer	4	3	12
Gli-3	ES 118/03	22	Internal granular layer	4	3	12
Gli-3	ES 112/03	21	External granular layer	3	3	9
Gli-3	ES 112/03	21	Molecular layer	3	3	9
Gli-3	ES 112/03	21	Internal granular layer	2	3	6
Gli-3	ES 67/03	28	External granular layer	4	3	12

Gli-3	ES 67/03	28	Molecular layer	4	3	12
Gli-3	ES 67/03	28	Internal granular layer	4	3	12
Gli-3	ES 67/03	28	External granular layer			
Gli-3	ES 67/03	28	Molecular layer			
Gli-3	ES 67/03	28	Internal granular layer			
Gli-3	ES 58/03	16	External granular layer	4	3	12
Gli-3	ES 58/03	16	Molecular layer	3	2	6
Gli-3	ES 58/03	16	Internal granular layer	2	2	4
Gli-3	ES 187/02	14	External granular layer			
Gli-3	ES 187/02	14	Molecular layer			
Gli-3	ES 187/02	14	Internal granular layer			
Gli-3	ES 102/02	13	External granular layer	4	3	12
Gli-3	ES 102/02	13	Molecular layer	4	3	12
Gli-3	ES 102/02	13	Internal granular layer	3	3	9
Gli-3	ES 38/02	17	External granular layer	3	3	9
Gli-3	ES 38/02	17	Molecular layer	2	3	6
Gli-3	ES 38/02	17	Internal granular layer	2	3	6
Gli-3	ES 23/02	19	External granular layer	3	3	9
Gli-3	ES 23/02	19	Molecular layer	2	3	6
Gli-3	ES 23/02	19	Internal granular layer	2	2	4
Gli-3	ES 22/02	19	External granular layer	3	3	9
Gli-3	ES 22/02	19	Molecular layer	4	3	12
Gli-3	ES 22/02	19	Internal granular layer	4	3	12
Gli-3	ES 15/02	21	External granular layer	4	3	12
Gli-3	ES 15/02	21	Molecular layer	3	3	9
Gli-3	ES 15/02	21	Internal granular layer	2	2	4
Gli-3	ES 4/02	19	External granular layer	3	2	6
Gli-3	ES 4/02	19	Molecular layer	2	2	4
Gli-3	ES 4/02	19	Internal granular layer	2	2	4

Gli-3	ES 154/01	18	External granular layer	3	2	6
Gli-3	ES 154/01	18	Molecular layer	2	2	4
Gli-3	ES 154/01	18	Internal granular layer	2	2	4
Gli-3	ES 151/01	13	External granular layer	4	2	8
Gli-3	ES 151/01	13	Molecular layer	2	2	4
Gli-3	ES 151/01	13	Internal granular layer	2	2	4
Gli-3	ES 129/01	26	External granular layer	4	2	8
Gli-3	ES 129/01	26	Molecular layer	2	3	6
Gli-3	ES 129/01	26	Internal granular layer	2	3	6
SHH	ES 49/04	20	External granular layer	3	2	6
SHH	ES 49/04	20	Molecular layer	3	2	6
SHH	ES 49/04	20	Internal granular layer	2	3	6
SHH	ES 15/04	16	External granular layer	3	3	9
SHH	ES 15/04	16	Molecular layer	2	2	4
SHH	ES 15/04	16	Internal granular layer	3	2	6
SHH	ES 3/04	14	External granular layer	3	3	9
SHH	ES 3/04	14	Molecular layer	3	2	6
SHH	ES 3/04	14	Internal granular layer	3	2	6
SHH	ES 155/03	12	External granular layer	4	3	12
SHH	ES 155/03	12	Molecular layer	2	2	4
SHH	ES 155/03	12	Internal granular layer	2	2	4
SHH	ES 148/03	22	External granular layer	3	2	6
SHH	ES 148/03	22	Molecular layer	2	2	4
SHH	ES 148/03	22	Internal granular layer	2	3	6
SHH	ES 144/03	13	External granular layer	4	3	12
SHH	ES 144/03	13	Molecular layer	2	2	4
SHH	ES 144/03	13	Internal granular layer	2	2	4
SHH	ES 142/03	14	External granular layer	4	3	12
SHH	ES 142/03	14	Molecular layer	1	2	2

SHH	ES 142/03	14	Internal granular layer	4	1	4
SHH	ES 128/03	18	External granular layer	3	2	6
SHH	ES 128/03	18	Molecular layer	2	2	4
SHH	ES 128/03	18	Internal granular layer	3	3	9
SHH	ES 118/03	22	External granular layer	3	2	6
SHH	ES 118/03	22	Molecular layer	2	2	4
SHH	ES 118/03	22	Internal granular layer	4	2	8
SHH	ES 112/03	21	External granular layer	2	2	4
SHH	ES 112/03	21	Molecular layer	2	2	4
SHH	ES 112/03	21	Internal granular layer	2	3	6
SHH	ES 67/03	28	External granular layer	3	2	6
SHH	ES 67/03	28	Molecular layer	2	2	4
SHH	ES 67/03	28	Internal granular layer	3	2	6
SHH	ES 67/03	28	External granular layer			
SHH	ES 67/03	28	Molecular layer			
SHH	ES 67/03	28	Internal granular layer			
SHH	ES 58/03	16	External granular layer	3	3	9
SHH	ES 58/03	16	Molecular layer	2	3	6
SHH	ES 58/03	16	Internal granular layer	3	3	9
SHH	ES 187/02	14	External granular layer			
SHH	ES 187/02	14	Molecular layer			
SHH	ES 187/02	14	Internal granular layer			
SHH	ES 102/02	13	External granular layer	4	3	12
SHH	ES 102/02	13	Molecular layer	2	3	6
SHH	ES 102/02	13	Internal granular layer	3	3	9
SHH	ES 38/02	17	External granular layer	4	3	12
SHH	ES 38/02	17	Molecular layer	3	2	6
SHH	ES 38/02	17	Internal granular layer	2	3	6
SHH	ES 23/02	19	External granular layer	4	3	12

SHH	ES 23/02	19	Molecular layer	3	2	6
SHH	ES 23/02	19	Internal granular layer	3	3	9
SHH	ES 22/02	19	External granular layer	4	2	8
SHH	ES 22/02	19	Molecular layer	3	2	6
SHH	ES 22/02	19	Internal granular layer	3	2	6
SHH	ES 15/02	21	External granular layer	4	3	12
SHH	ES 15/02	21	Molecular layer	3	2	6
SHH	ES 15/02	21	Internal granular layer	3	3	9
SHH	ES 4/02	19	External granular layer	4	3	12
SHH	ES 4/02	19	Molecular layer	4	2	8
SHH	ES 4/02	19	Internal granular layer	4	2	8
SHH	ES 154/01	18	External granular layer	4	3	12
SHH	ES 154/01	18	Molecular layer	3	2	6
SHH	ES 154/01	18	Internal granular layer	3	2	6
SHH	ES 151/01	13	External granular layer			
SHH	ES 151/01	13	Molecular layer			
SHH	ES 151/01	13	Internal granular layer			
SHH	ES 129/01	26	External granular layer	4	3	12
SHH	ES 129/01	26	Molecular layer	3	2	6
SHH	ES 129/01	26	Internal granular layer	3	3	9
patched	ES 49/04	20	External granular layer	4	2	8
patched	ES 49/04	20	Molecular layer	3	2	6
patched	ES 49/04	20	Internal granular layer	3	2	6
patched	ES 15/04	16	External granular layer	3	2	6
patched	ES 15/04	16	Molecular layer	3	2	6
patched	ES 15/04	16	Internal granular layer	2	2	4
patched	ES 3/04	14	External granular layer	3	2	6
patched	ES 3/04	14	Molecular layer	2	2	4
patched	ES 3/04	14	Internal granular layer	2	2	4

patched	ES 155/03	12	External granular layer	4	3	12
patched	ES 155/03	12	Molecular layer	2	2	4
patched	ES 155/03	12	Internal granular layer	3	2	6
patched	ES 148/03	22	External granular layer	3	3	9
patched	ES 148/03	22	Molecular layer	2	2	4
patched	ES 148/03	22	Internal granular layer	3	3	9
patched	ES 144/03	13	External granular layer	4	2	8
patched	ES 144/03	13	Molecular layer	2	2	4
patched	ES 144/03	13	Internal granular layer	3	2	6
patched	ES 142/03	14	External granular layer	4	2	8
patched	ES 142/03	14	Molecular layer	2	2	4
patched	ES 142/03	14	Internal granular layer	2	2	4
patched	ES 128/03	18	External granular layer	2	3	6
patched	ES 128/03	18	Molecular layer	2	3	6
patched	ES 128/03	18	Internal granular layer	3	3	9
patched	ES 118/03	22	External granular layer	4	2	8
patched	ES 118/03	22	Molecular layer	3	2	6
patched	ES 118/03	22	Internal granular layer	3	2	6
patched	ES 112/03	21	External granular layer	4	2	8
patched	ES 112/03	21	Molecular layer	2	3	6
patched	ES 112/03	21	Internal granular layer	3	3	9
patched	ES 67/03	28	External granular layer	4	2	8
patched	ES 67/03	28	Molecular layer	2	2	4
patched	ES 67/03	28	Internal granular layer	3	2	6
patched	ES 67/03	28	External granular layer			
patched	ES 67/03	28	Molecular layer			
patched	ES 67/03	28	Internal granular layer			
patched	ES 58/03	16	External granular layer	3	2	6
patched	ES 58/03	16	Molecular layer	2	2	4

patched	ES 58/03	16	Internal granular layer	3	2	6
patched	ES 187/02	14	External granular layer			
patched	ES 187/02	14	Molecular layer			
patched	ES 187/02	14	Internal granular layer			
patched	ES 102/02	13	External granular layer	4	1	4
patched	ES 102/02	13	Molecular layer	2	2	4
patched	ES 102/02	13	Internal granular layer	3	2	6
patched	ES 38/02	17	External granular layer	3	3	9
patched	ES 38/02	17	Molecular layer	3	2	6
patched	ES 38/02	17	Internal granular layer	2	3	6
patched	ES 23/02	19	External granular layer	3	2	6
patched	ES 23/02	19	Molecular layer	2	2	4
patched	ES 23/02	19	Internal granular layer	3	3	9
patched	ES 22/02	19	External granular layer	3	2	6
patched	ES 22/02	19	Molecular layer	2	2	4
patched	ES 22/02	19	Internal granular layer	3	2	6
patched	ES 15/02	21	External granular layer	4	3	12
patched	ES 15/02	21	Molecular layer	2	3	6
patched	ES 15/02	21	Internal granular layer	3	3	9
patched	ES 4/02	19	External granular layer	3	3	9
patched	ES 4/02	19	Molecular layer	4	2	8
patched	ES 4/02	19	Internal granular layer	3	2	6
patched	ES 154/01	18	External granular layer	4	3	12
patched	ES 154/01	18	Molecular layer	2	3	6
patched	ES 154/01	18	Internal granular layer	4	3	12
patched	ES 151/01	13	External granular layer	4	3	12
patched	ES 151/01	13	Molecular layer	2	3	6
patched	ES 151/01	13	Internal granular layer	2	3	6
patched	ES 129/01	26	External granular layer	3	2	6

patched	ES 129/01	26	Molecular layer	3	2	6
patched	ES 129/01	26	Internal granular layer	1	3	3
Smoothened	ES 49/04	20	External granular layer	4	1	4
Smoothened	ES 49/04	20	Molecular layer	3	1	3
Smoothened	ES 49/04	20	Internal granular layer	3	1	3
Smoothened	ES 15/04	16	External granular layer	3	3	9
Smoothened	ES 15/04	16	Molecular layer	2	2	4
Smoothened	ES 15/04	16	Internal granular layer	2	2	4
Smoothened	ES 3/04	14	External granular layer	4	2	8
Smoothened	ES 3/04	14	Molecular layer	2	1	2
Smoothened	ES 3/04	14	Internal granular layer	3	1	3
Smoothened	ES 155/03	12	External granular layer			
Smoothened	ES 155/03	12	Molecular layer			
Smoothened	ES 155/03	12	Internal granular layer			
Smoothened	ES 148/03	22	External granular layer			
Smoothened	ES 148/03	22	Molecular layer			
Smoothened	ES 148/03	22	Internal granular layer	1	1	1
Smoothened	ES 144/03	13	External granular layer	4	2	8
Smoothened	ES 144/03	13	Molecular layer	3	2	6
Smoothened	ES 144/03	13	Internal granular layer	3	2	6
Smoothened	ES 142/03	14	External granular layer	4	2	8
Smoothened	ES 142/03	14	Molecular layer	3	1	3
Smoothened	ES 142/03	14	Internal granular layer	3	1	3
Smoothened	ES 128/03	18	External granular layer	3	2	6
Smoothened	ES 128/03	18	Molecular layer	2	2	4
Smoothened	ES 128/03	18	Internal granular layer	2	2	4
Smoothened	ES 118/03	22	External granular layer	4	2	8
Smoothened	ES 118/03	22	Molecular layer	2	2	4
Smoothened	ES 118/03	22	Internal granular layer	3	2	6

Smoothened	ES 112/03	21	External granular layer	4	3	12
Smoothened	ES 112/03	21	Molecular layer	2	2	4
Smoothened	ES 112/03	21	Internal granular layer	3	2	6
Smoothened	ES 67/03	28	External granular layer	3	3	9
Smoothened	ES 67/03	28	Molecular layer	3	2	6
Smoothened	ES 67/03	28	Internal granular layer	3	2	6
Smoothened	ES 67/03	28	External granular layer			
Smoothened	ES 67/03	28	Molecular layer			
Smoothened	ES 67/03	28	Internal granular layer			
Smoothened	ES 58/03	16	External granular layer	3	2	6
Smoothened	ES 58/03	16	Molecular layer	2	1	2
Smoothened	ES 58/03	16	Internal granular layer	2	1	2
Smoothened	ES 187/02	14	External granular layer			
Smoothened	ES 187/02	14	Molecular layer			
Smoothened	ES 187/02	14	Internal granular layer			
Smoothened	ES 102/02	13	External granular layer	4	2	8
Smoothened	ES 102/02	13	Molecular layer	2	2	4
Smoothened	ES 102/02	13	Internal granular layer	3	2	6
Smoothened	ES 38/02	17	External granular layer	4	3	12
Smoothened	ES 38/02	17	Molecular layer	3	2	6
Smoothened	ES 38/02	17	Internal granular layer	3	2	6
Smoothened	ES 23/02	19	External granular layer	3	2	6
Smoothened	ES 23/02	19	Molecular layer	2	2	4
Smoothened	ES 23/02	19	Internal granular layer	3	3	9
Smoothened	ES 22/02	19	External granular layer	4	2	8
Smoothened	ES 22/02	19	Molecular layer	2	2	4
Smoothened	ES 22/02	19	Internal granular layer	3	2	6
Smoothened	ES 15/02	21	External granular layer	4	2	8
Smoothened	ES 15/02	21	Molecular layer	3	2	6

Smoothened	ES 15/02	21	Internal granular layer	2	2	4
Smoothened	ES 4/02	19	External granular layer	4	2	8
Smoothened	ES 4/02	19	Molecular layer	2	2	4
Smoothened	ES 4/02	19	Internal granular layer	3	2	6
Smoothened	ES 154/01	18	External granular layer	4	2	8
Smoothened	ES 154/01	18	Molecular layer	3	2	6
Smoothened	ES 154/01	18	Internal granular layer	2	2	4
Smoothened	ES 151/01	13	External granular layer	4	2	8
Smoothened	ES 151/01	13	Molecular layer	2	2	4
Smoothened	ES 151/01	13	Internal granular layer	3	2	6
Smoothened	ES 129/01	26	External granular layer	4	3	12
Smoothened	ES 129/01	26	Molecular layer	2	2	4
Smoothened	ES 129/01	26	Internal granular layer	2	2	4

Supplementary Table 3: Comparison of multi-scores/region in the telencephalon assessed by likelihood-ratio test (*not significant after Bonferroni-Holm correction).

A	B	p-value
Shh – MZ	Shh – CP	<0.0001
Shh – MZ	Shh – SP	0.0048
Shh – MZ	Shh – IZ	0.4131
Shh – MZ	Shh – SVZ	0.3040
Shh – MZ	Shh – VZ	0.0014
Shh – CP	Shh – SP	0.0303*
Shh – CP	Shh – IZ	<0.0001
Shh – CP	Shh – SVZ	0.0026
Shh – CP	Shh – VZ	0.1971
Shh – SP	Shh – IZ	0.0006
Shh – SP	Shh – SVZ	0.2578
Shh – SP	Shh – VZ	0.2335
Shh – IZ	Shh – SVZ	0.2670
Shh – IZ	Shh – VZ	0.0003
Shh – SVZ	Shh – VZ	0.0475*
Ptch – MZ	Ptch – CP	0.1239
Ptch – MZ	Ptch – SP	0.2131
Ptch – MZ	Ptch – IZ	0.3432
Ptch – MZ	Ptch – SVZ	0.0409*
Ptch – MZ	Ptch – VZ	0.0024
Ptch – CP	Ptch – SP	0.2064
Ptch – CP	Ptch – IZ	0.0822
Ptch – CP	Ptch – SVZ	0.0486*
Ptch – CP	Ptch – VZ	0.3338
Ptch – SP	Ptch – IZ	0.3723
Ptch – SP	Ptch – SVZ	0.1044
Ptch – SP	Ptch – VZ	0.0095*
Ptch – IZ	Ptch – SVZ	0.6537
Ptch – IZ	Ptch – VZ	0.0029
Ptch – SVZ	Ptch – VZ	0.0012
Smo – MZ	Smo – CP	0.0022
Smo – MZ	Smo – SP	0.0034
Smo – MZ	Smo – IZ	0.7346
Smo – MZ	Smo – SVZ	0.23
Smo – MZ	Smo – VZ	0.0008
Smo – CP	Smo – SP	0.1158
Smo – CP	Smo – IZ	0.0041
Smo – CP	Smo – SVZ	0.1432
Smo – CP	Smo – VZ	0.6520
Smo – SP	Smo – IZ	0.0075*
Smo – SP	Smo – SVZ	0.1357
Smo – SP	Smo – VZ	0.0064*
Smo – IZ	Smo – SVZ	0.2552
Smo – IZ	Smo – VZ	0.0008
Smo – SVZ	Smo – VZ	0.0236

A	B	p-value
Gli1 – MZ	Gli1 – CP	0.0116*
Gli1 – MZ	Gli1 – SP	0.2734
Gli1 – MZ	Gli1 – IZ	0.8151
Gli1 – MZ	Gli1 – SVZ	0.2131
Gli1 – MZ	Gli1 – VZ	0.2511
Gli1 – CP	Gli1 – SP	0.2433
Gli1 – CP	Gli1 – IZ	0.0025
Gli1 – CP	Gli1 – SVZ	0.0636
Gli1 – CP	Gli1 – VZ	0.2289
Gli1 – SP	Gli1 – IZ	0.0811
Gli1 – SP	Gli1 – SVZ	0.0725
Gli1 – SP	Gli1 – VZ	0.4272
Gli1 – IZ	Gli1 – SVZ	0.3232
Gli1 – IZ	Gli1 – VZ	0.2792
Gli1 – SVZ	Gli1 – VZ	0.8255
Gli2 – MZ	Gli2 – CP	<0.0001
Gli2 – MZ	Gli2 – SP	0.0075*
Gli2 – MZ	Gli2 – IZ	0.6665
Gli2 – MZ	Gli2 – SVZ	0.0452*
Gli2 – MZ	Gli2 – VZ	0.0274*
Gli2 – CP	Gli2 – SP	0.0393*
Gli2 – CP	Gli2 – IZ	<0.0001
Gli2 – CP	Gli2 – SVZ	<0.0001
Gli2 – CP	Gli2 – VZ	0.0966
Gli2 – SP	Gli2 – IZ	0.0063*
Gli2 – SP	Gli2 – SVZ	0.0004
Gli2 – SP	Gli2 – VZ	0.8373
Gli2 – IZ	Gli2 – SVZ	0.1102
Gli2 – IZ	Gli2 – VZ	0.0198*
Gli2 – SVZ	Gli2 – VZ	0.0017
Gli3 – MZ	Gli3 – CP	0.024*
Gli3 – MZ	Gli3 – SP	0.074*
Gli3 – MZ	Gli3 – IZ	0.01*
Gli3 – MZ	Gli3 – SVZ	0.2767
Gli3 – MZ	Gli3 – VZ	0.0877
Gli3 – CP	Gli3 – SP	0.2942
Gli3 – CP	Gli3 – IZ	0.0248*
Gli3 – CP	Gli3 – SVZ	0.1206
Gli3 – CP	Gli3 – VZ	0.1365
Gli3 – SP	Gli3 – IZ	0.5938
Gli3 – SP	Gli3 – SVZ	0.3744
Gli3 – SP	Gli3 – VZ	0.0403*
Gli3 – IZ	Gli3 – SVZ	0.1693
Gli3 – IZ	Gli3 – VZ	0.0044*
Gli3 – SVZ	Gli3 – VZ	0.023*

Supplementary Table 4: Comparison of multi-scores/region in the cerebellum

A	B	p-value
Shh – EGL	Shh – ML	<0.0001
Shh – EGL	Shh – IGL	0.0016
Shh – ML	Shh – IGL	0.0143
Ptch – EGL	Ptch – ML	<0.0001
Ptch – EGL	Ptch – IGL	0.0151
Ptch – ML	Ptch – IGL	0.0099
Smo – EGL	Smo – ML	<0.0001
Smo – EGL	Smo – IGL	<0.0001
Smo – ML	Smo – IGL	0.2923
Gli1 – EGL	Gli1 – ML	0.0027
Gli1 – EGL	Gli1 – IGL	0.0581
Gli1 – ML	Gli1 – IGL	<0.0001
Gli2 – EGL	Gli2 – ML	<0.0001
Gli2 – EGL	Gli2 – IGL	0.0021
Gli2 – ML	Gli2 – IGL	0.0023
Gli3 – EGL	Gli3 – ML	0.0052
Gli3 – EGL	Gli3 – IGL	0.0006
Gli3 – ML	Gli3 – IGL	0.5789
